# Beta-blocker therapy in patients with COPD: a systematic literature review and meta-analysis with multiple treatment comparison

**DOI:** 10.1186/s12931-021-01661-8

**Published:** 2021-02-23

**Authors:** Claudia Gulea, Rosita Zakeri, Vanessa Alderman, Alexander Morgan, Jack Ross, Jennifer K. Quint

**Affiliations:** 1 National Heart and Lung Institute, Imperial College London, Manresa Road, London, UK; 2 NIHR Imperial Biomedical Research Centre, London, UK; 3 British Heart Foundation Centre for Research Excellence, King’s College London, London, UK; 4 Homerton University Hospital NHS Foundation Trust, London, UK; 5 Epsom and St. Helier University Hospitals NHS Trust, Epsom, UK; 6 Guy’s & St Thomas’ NHS Foundation Trust, London, UK; 7 Royal Brompton & Harefield NHS Foundation Trust, London, UK

**Keywords:** COPD, Beta-blockers, Network meta-analysis

## Abstract

**Background:**

Beta-blockers are associated with reduced mortality in patients with cardiovascular disease but are often under prescribed in those with concomitant COPD, due to concerns regarding respiratory side-effects. We investigated the effects of beta-blockers on outcomes in patients with COPD and explored within-class differences between different agents.

**Methods:**

We searched the Cochrane Central Register of Controlled Trials, Embase, Cumulative Index to Nursing and Allied Health Literature (CINAHL) and Medline for observational studies and randomized controlled trials (RCTs) investigating the effects of beta-blocker exposure versus no exposure or placebo, in patients with COPD, with and without cardiovascular indications. A meta-analysis was performed to assess the association of beta-blocker therapy with acute exacerbations of COPD (AECOPD), and a network meta-analysis was conducted to investigate the effects of individual beta-blockers on FEV1. Mortality, all-cause hospitalization, and quality of life outcomes were narratively synthesized.

**Results:**

We included 23 observational studies and 14 RCTs. In pooled observational data, beta-blocker therapy was associated with an overall reduced risk of AECOPD versus no therapy (HR 0.77, 95%CI 0.70 to 0.85)*.* Among individual beta-blockers, only propranolol was associated with a relative reduction in FEV1 versus placebo, among 199 patients evaluated in RCTs. Narrative syntheses on mortality, all-cause hospitalization and quality of life outcomes indicated a high degree of heterogeneity in study design and patient characteristics but suggested no detrimental effects of beta-blocker therapy on these outcomes.

**Conclusion:**

The class effect of beta-blockers remains generally positive in patients with COPD. Reduced rates of AECOPD, mortality, and improved quality of life were identified in observational studies, while propranolol was the only agent associated with a deterioration of lung function in RCTs.

## Background

COPD and cardiovascular disease (CVD) often co-occur, in an interaction characterized by complex biological mechanisms and risk factors such as smoking. Beta-blockers are recommended in treatment regimens of people with heart failure (HF), following myocardial infarction (MI), angina or hypertension, due to proven mortality benefits [1–4]. Seventeen years after the publication of the first robust meta-analysis demonstrating that beta-blockers do not impair lung function in patients with COPD [5], prescription rates remain lower than for people without COPD, among those with an indication for treatment. This treatment gap is thought to be, in part, due to concerns regarding adverse respiratory effects (such as a decrease in lung function) despite accumulating evidence to the contrary[6]. Concomitant CVD independently affects mortality and hospitalization in patients with COPD, further adding to the clinical burden and complexity of treatment pathways in these patients[7, 8].

COPD guidelines recommend the use of cardioselective beta-blockers when appropriate, reinforced by evidence gathered in a Cochrane review [9]. Data regarding the association of beta-blocker therapy with mortality and acute exacerbations due to COPD (AECOPD) is derived mostly from observational data and previous reviews have aggregated results for cardio and non-cardioselective agents [10, 11]. However, a recent single RCT [12] reported more hospitalizations due to AECOPD in patients treated with metoprolol as compared to placebo, though results on mortality and FEV1 were inconclusive.

Our study expands on previous literature by dissecting the effects of beta-blockers from both RCTs and observational studies, on a wide-range of clinically-relevant end points (mortality, AECOPD, FEV1, all-cause hospitalization and quality of life outcomes such as St. George’s Respiratory Questionnaire (SGRQ), 12 and 6MWT (12, 6 Minute Walking Test) and the Short-Form Health Survey Questionnaire (SF-36), thereby providing a comprehensive assessment of the effects of beta-blocker treatment in COPD. We have two overarching aims: (1) to identify and assess the class-effect of beta-blockers and (2) to compare within-class effects of beta-blockers on the aforementioned outcomes. If all studies have a minimum of one intervention in common with another, it will be possible to create a network of treatments, allowing both direct and indirect evidence to be used in deriving comparisons between beta-blockers not studied in a head-to-head manner, using a network-meta-analysis (NMA). Importantly, we also want to address a current gap in knowledge—we will investigate whether the potential benefits of beta-blockers are limited to those with CVD or may extend in the wider COPD population with or without undiagnosed CVD.

## Methods

The protocol for this review was previously published [13]. Searches were conducted from inception to January 2021 in MEDLINE, Embase and CINAHL via Ovid and The Cochrane Collection Central Register of Clinical Trials to identify studies that examined the association between beta-blockers in patients with COPD (defined as post-bronchodilator FEV1/FVC of < 0.70, or as being in accordance with GOLD guidelines [6]; patients with a clinical diagnosis of COPD) and clinical, safety and quality of life outcomes. To ensure we captured all relevant evidence, we included prospective interventional trials (RCTs) and prospective observational studies (single-arm studies were excluded). At the screening stage, due to a scarcity of prospective observational studies, we decided to also include retrospective observational studies. We required all studies to report on mortality, AECOPD, all-cause hospitalization and quality of life outcomes. We also manually searched reference lists of previously published reviews. Abstracts were screened for inclusion by two independent reviewers, with any discrepancies resolved through discussion. Full texts of included abstracts were screened by a single investigator, and 25% of articles were additionally validated by a second investigator. Full inclusion/exclusion criteria applied at each stage are available in the Additional file 1: Table S1.

### Data extraction and quality assessment

For each accepted study, data was extracted on design, characteristics of study population including comorbidities, inclusion and exclusion criteria, treatment administered and the reported effect of beta-blocker on included outcomes. Details on planned data extraction are available in the protocol [13]. Authors were contacted to clarify ambiguously reported data from published reports. Included observational studies were assessed for risk of bias using the ROBIN-I [14] tool for cohort studies and RCTs were assessed using the ROB tool [15]. Bias domains evaluated include confounding, reporting, attrition, or measurement of outcomes. Each domain was assigned to a risk category such as “low”, “moderate”, “high” or “unclear” for observational studies and “low”, “high” or “some concerns” for RCTs. Additionally, we assessed the certainty of the evidence using the Grading of Recommendations Assessment Development and Evaluation (GRADE) framework [16].

### Endpoints

Searches identified studies reporting on all-cause mortality, AECOPD, FEV1, all-cause hospitalization, SGRQ, the 12 and 6 MWT, and the SF-36. Four researchers extracted data from the included articles, and all were validated by a second researcher.

### Data analysis

Where included studies were reasonably statistically and clinically similar, we pooled results using meta-analysis (to investigate class-effect of beta-blocker treatment), or NMA, where data on individual therapeutic compounds was available. Publication bias was assessed using funnel plots if there were at least 10 studies included in meta-analysis [17]. For binary outcomes we initially included studies that reported on outcomes in any format (Hazard ratio [HR], Odds Ratio [OR], Risk ratio, Incidence Rate); however, the final inclusion list contains only studies reporting HRs since this was the most common amongst included studies. Heterogeneity was assessed using I^2^ [18].

### FEV1—Network meta-analysis of RCTs

We performed a random-effects Bayesian NMA to estimate mean change in FEV1 between patients who received individual beta-blockers versus (vs.) placebo with 95% Credible intervals (CrI), using package gemtc [19] in R v3.6. CrIs represent the 95% probability that the true underlying effect lies in the interval specified. In cases where the standard deviation (SD) for the FEV1 measures was not reported, the SD was extrapolated by averaging the SDs from other studies with similar sample characteristics. Random-effect analyses are widely accepted as the appropriate, more conservative approach when there is heterogeneity across study methods. By contrast, fixed-effect models assume that effect size associated with an intervention does not vary from study to study, and they may be particularly appropriate when only few studies are available for analysis. The best model fit for each network was selected based on a review of the deviance information criterion (DIC) and an evaluation of the different model assumptions.

NMAs include direct and indirect evidence from trials to determine the best available treatment with respect to an outcome of interest. For the results to be valid, NMA assumptions need to be met, including the transitivity and consistency assumptions. For the transitivity assumption to be met, the studies that contribute direct evidence must be similar in distribution of covariates and effect modifiers across the trial populations. Inconsistency occurs when the indirect evidence in a network is different compared to the direct evidence. Assessing consistency of data in the network model is done implicitly in package “gemtc” which uses a decision rule to choose which comparisons may be potentially inconsistent—the “node-splitting” method. Small study effects were explored by conducting comparison-adjusted funnel plots [20] and publication bias was assessed by Egger’s test among comparisons of beta-blockers and placebo. A value of p < 0.1 indicated significant publication bias. To assess the probability that a treatment is the best within a network, rank probabilities were determined—the probability for each treatment to obtain each possible rank in terms of their relative effects. Interpretation needs to be made with caution, because a treatment may have a high probability of being first, or last treatment and its’ benefit over other treatments may be of little clinical value [21]. For this reason, we report a full ranking profile (where each treatment is assigned a probability of being first, second, and so on, best treatment in the network) which was derived using the surface under the cumulative ranking curve (SUCRA) [22].

### Sensitivity analyses

We conducted two meta-regressions to establish whether FEV1 measurement at baseline or study duration influenced the main NMA results. These variables were added, separately, as covariates in the main NMA model; FEV1 as a continuous variable and follow-up dichotomised into short follow-up (less than 24 h) vs. long follow-up (more than 24 h). We compared model fit between models with and without covariates using the DIC. Where possible, we analyzed patients with and without CVD separately.

### AECOPD—meta-analysis of observational studies

We pooled HRs denoting the association between beta-blocker exposure (vs. no exposure) amongst patients with COPD, using random-effects meta-analysis with the DerSimonian-Lard estimator in “metafor” [23] package in R v3.6.

### Mortality; quality of life—narrative synthesis

If studies were too heterogeneous (I^2^ > 75%), or where outcomes were reported in under three studies per treatment comparison, quantitative analysis was not reported, but summary results were graphed on forest plots without pooling the results (mortality) and/or synthesized qualitatively (quality of life outcomes).

## Results

**Fig. 1:**
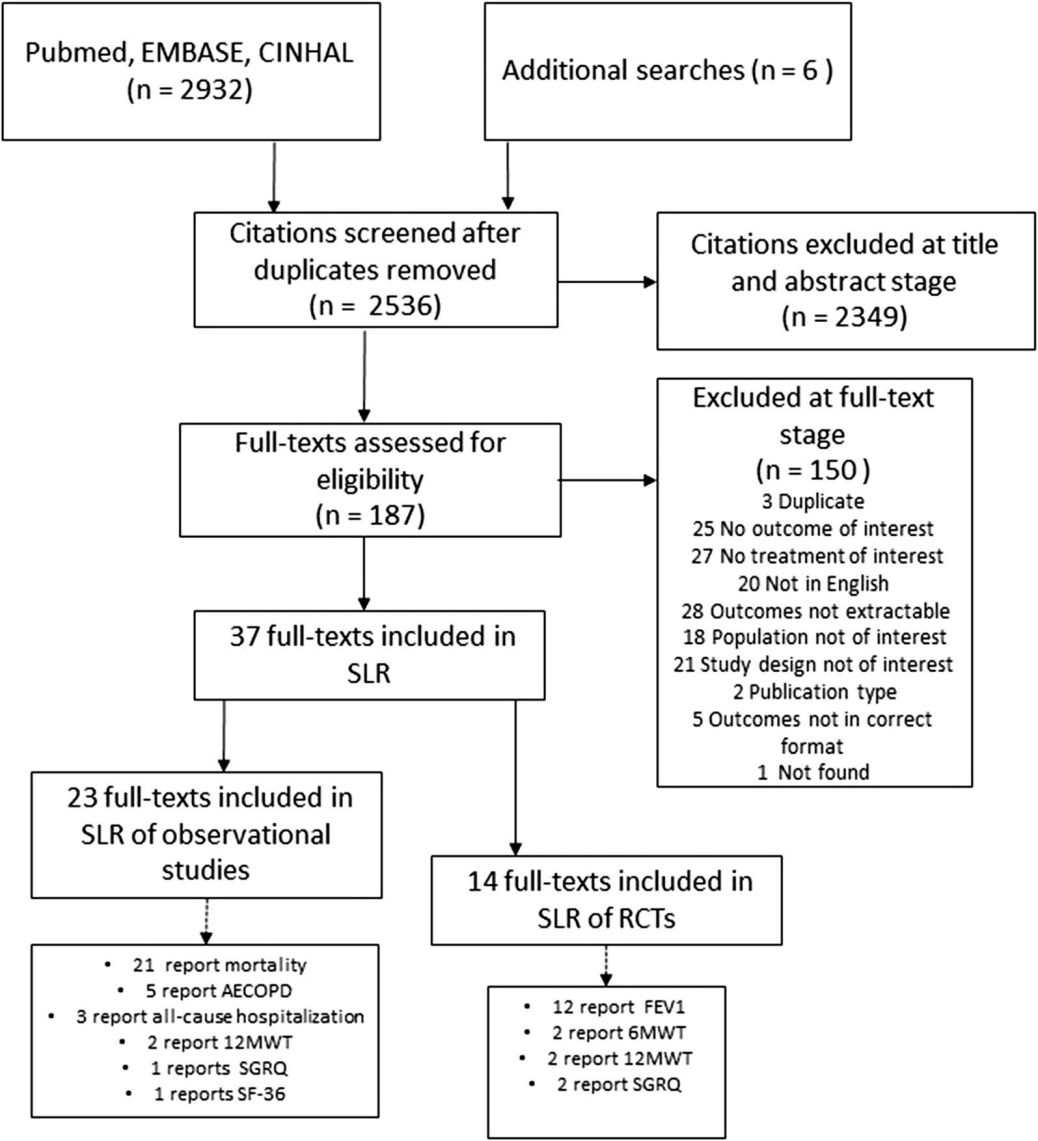
PRISMA

The database search identified 2932 potentially relevant articles whilst other sources revealed six. After title and abstract screening, 187 articles underwent full-text review. We included 23 observational studies and 14 RCTs that reported on patients with COPD, in the systematic literature review. Out of a total of 23 observational studies, 21 reported on mortality [24–44], five reported on AECOPD [24, 33, 35, 45, 46] three reported on all-cause hospitalization [47–49], one reported on SGRQ [45] and one reported on SF-36 [42]. From a total of 14 RCTs, 12 reported on FEV1 [50–61], two each reported on 12MWT [59, 62] and 6MWT [12, 56] and two reported on SGRQ [12, 56] (Fig. 1).

### AECOPD

According to our protocol, we intended to include data on effect of beta-blockers on AECOPD from RCTs, however our search strategy revealed only one study of this kind [12]. Based on a population of 532 patients with moderate to severe COPD, the authors reported no significant difference in time to first AECOPD (of any severity) between metoprolol and placebo, however the use of the beta-blocker was associated with a higher risk of severe exacerbation (requiring hospitalization). This study could not be included in the quantitative analysis, as there was no other RCT data to corroborate.

### Quantitative analyses

There were five [24, 33, 35, 45, 46] observational studies reporting on the effect of beta-blockers on AECOPD in patients from at least five countries across Europe. Follow-up varied from 0.76 [46] to 7.2 years [33]. The average age of the patients ranged from 62.8 [24] to 74 [46] years old and the proportion of males from 49.8% [33] to 72.3% [45]. Only two studies reported on smoking status [33, 45], which indicated the majority of patients were either current or former smokers. Comorbidities were frequent in all cohorts, specifically CVD, reported in all but one study [35]. Body mass index (BMI) was reported in only two studies and ranged between 25.5 [45] and 29. 9 kg/m^2^ [24]. All study characteristics are available in Additional file 1: Table S2 and Table S3.

**Fig. 2:**
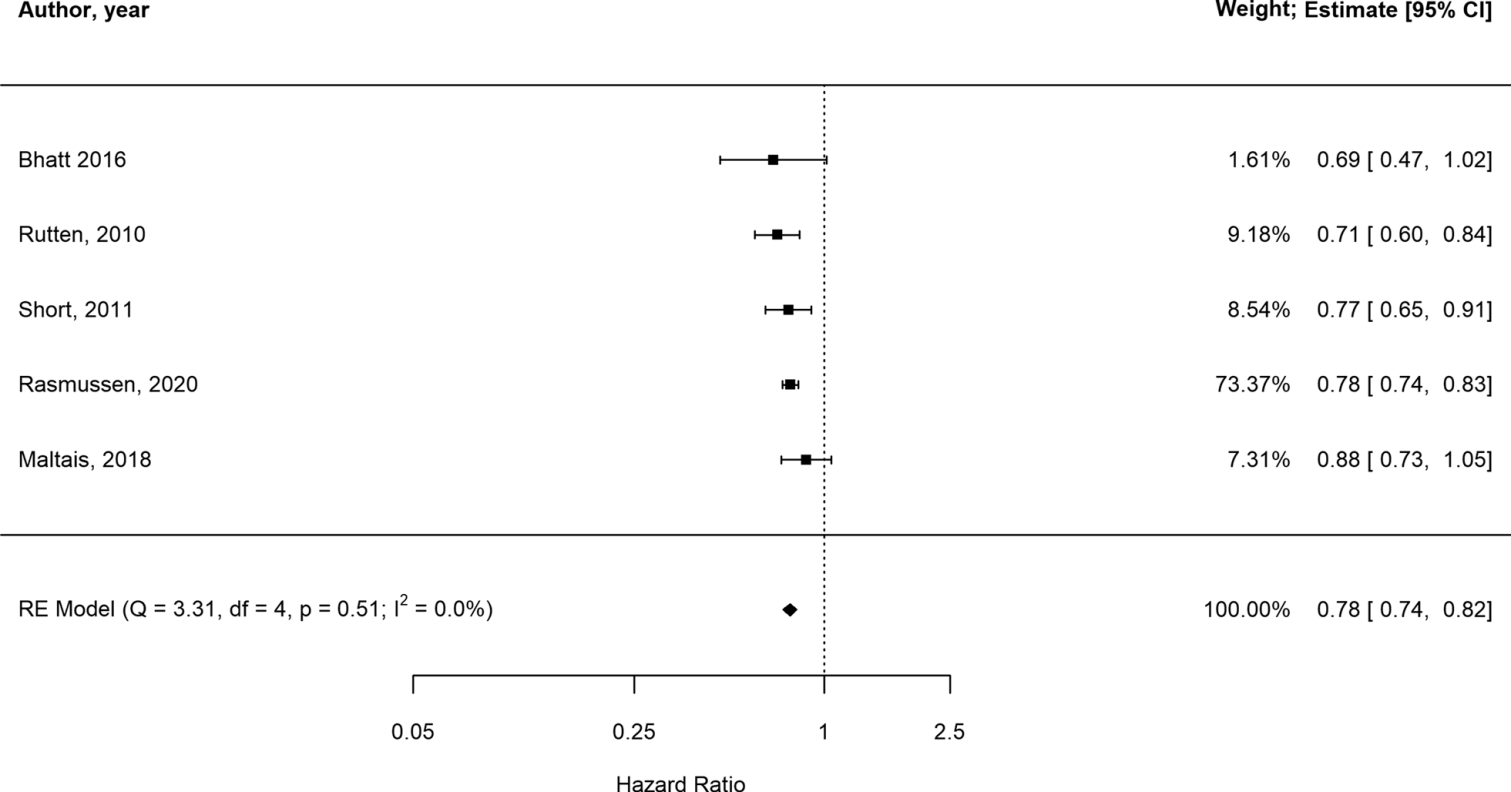
Forest plot illustrating results of the meta-analysis evaluating the impact of beta-blocker therapy versus no beta-blocker therapy on AECOPD in patients with COPD (Estimate: *HR* hazard ratio, *95% CI* confidence interval)

In the presence of low statistical heterogeneity (< 25%), the random effects and fixed effects method for pooling effect estimates give identical results. Due to low heterogeneity (I^2^ = 0, owing to the large weight attributed to one study only [46]) and the small overall number of studies, we report both random and fixed-effects meta-analyses of AECOPD. In random-effects analysis, the pooled estimate risk of AECOPD associated with beta-blocker use, from an total of 27,717 patients, was HR 0.78 [95%CI 0.74–0.82] suggesting a reduction in relative risk in the presence of beta-blockers (Fig. 2, Additional file 1: Table S4 for individual study outcomes). The fixed-effects meta-analysis yielded similar results (Additional File 1: Figure S1). Due to low number of studies we could not formally assess the extent of publication bias. The GRADE assessment indicated the overall quality of evidence based on which the meta-analysis was conducted was low (Additional file 1: Table S18).

### FEV1

Data from 12 RCTs evaluating FEV1 in 199 patients and seven beta-blockers (atenolol, bisoprolol, carvedilol, celiprolol, metoprolol, propranolol, labetalol) were evaluated [50–55, 57–61, 63]. Duration of trials varied from 1 hour [53, 59] to 3–4 months [57] and FEV1 measurement at baseline between 1.15 [59] and 2.41 L [l][61]. Most patients were over 40 years old except for one study where mean age was 39 [60]. Across all studies, over 50% of the patient population were male and four studies only included patients with CVD or hypertension explicitly [50, 54, 55, 57] (Additional file 1: Table S5). A comparison between studies enrolling patients with CVD and those enrolling patients with COPD only is difficult due to scarcity of reported data. BMI was available in two studies of COPD and CVD [55, 57] and in one study only which excluded CVD [58]. Estimates were however similar and denoted overweight, but not obese patient populations. Celiprolol was the only treatment which was evaluated in patients without CVD exclusively, in one trial [61] only. Sample size, age and proportion of males were similar across all studies.

**Fig. 3:**
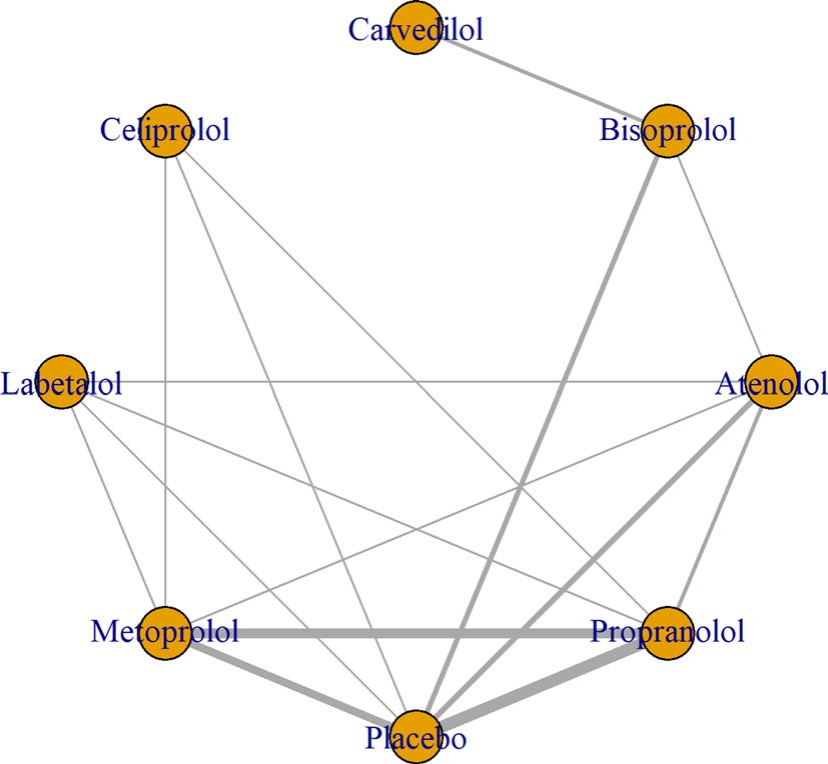
Network of beta-blockers used to treat patients with COPD, from RCTs assessing FEV1

**Fig. 4:**
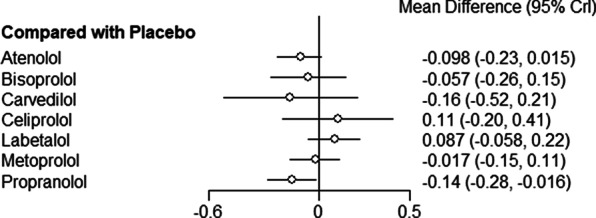
Network meta-analysis results for mean difference in FEV1 (95 CrI), beta-blockers compared to placebo [measured in liters, *CrI* credible intervals]

Figure 3 shows the network of eligible comparisons for FEV1 mean change from baseline to time-point, including seven treatments. All beta-blockers except carvedilol were evaluated in at least one placebo-controlled trial. Individual study FEV1 measurements are presented in Additional file 1: Table S6. Figure 4 and Additional file 1: Table S7 show the NMA results for FEV1. Consistency results are illustrated in Additional file 1: Figure S2. Effects relative to placebo are presented separately for each treatment.

**Fig. 5:**
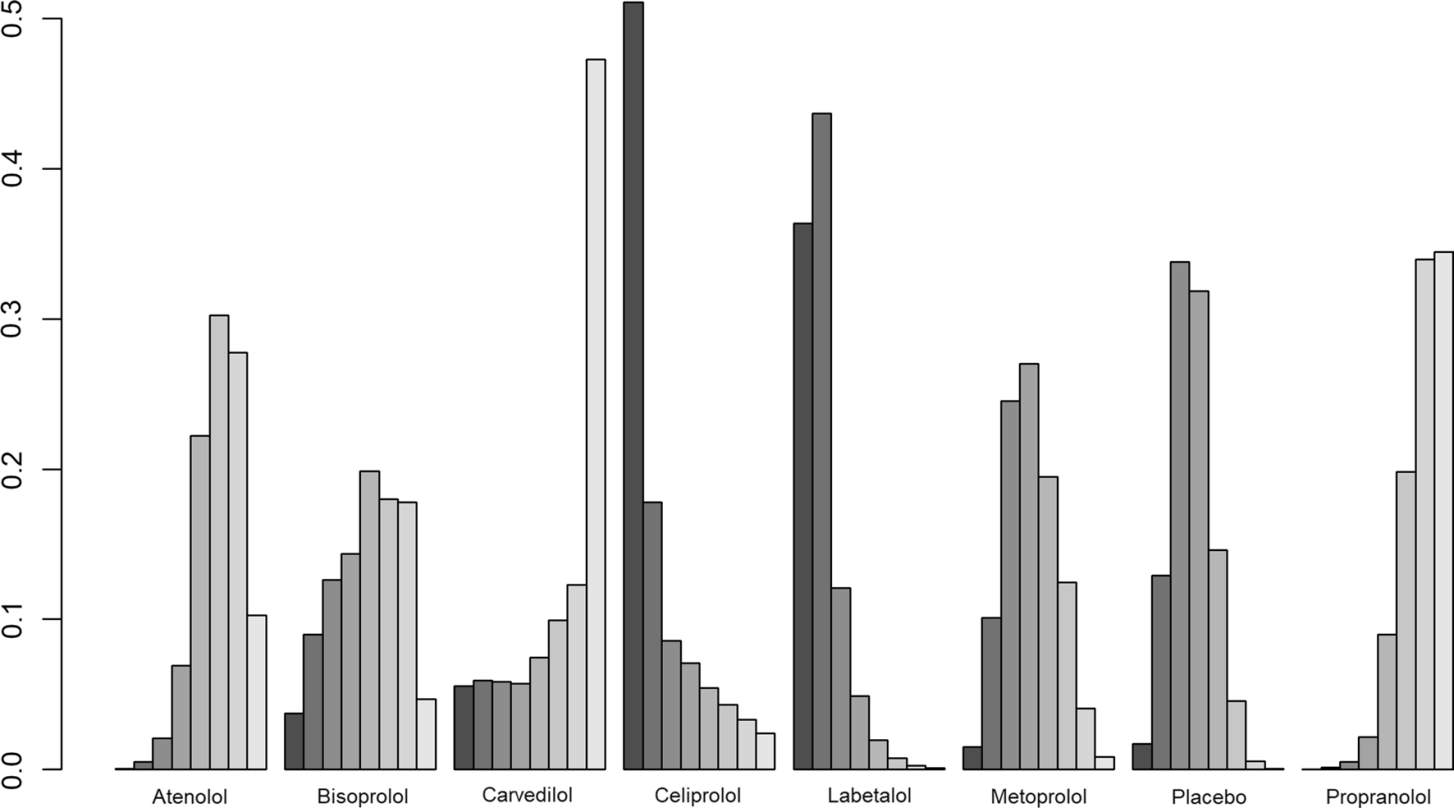
Rankogram illustrating probabilities that each treatment is first, second, third…eighth with regards to FEV1 improvement

There was no significant difference in FEV1 amongst all beta-blockers except for propranolol, which was the only treatment associated with a decrease in FEV1 (mean difference [MD]:− 0.14 ml, 95% CrI,  0.28 to 0.016). Individual medications were ranked and are presented with estimates of the probability that each is the best treatment (i.e. probability that the treatment improves lung function). Figure 5 shows that celiprolol had the highest likelihood of being ranked best treatment, followed by labetalol. For the second rank, the same treatments appear the most likely. Overall, the SUCRA results based on the rankogram values appear to suggest labetalol (86.2%) and celiprolol (80%) are the most likely of being the best treatments to positively affect FEV1, whilst propranolol was the least likely (16.2% probability of being the best) (Additional file 1: Table S9). According to the comparison-adjusted funnel plot, no publication bias was found for Egger’s test (p = 0.1286, Additional file 1: Figure S3).

### Sensitivity analyses

The meta-regression analyses, with baseline FEV1 measurement, follow-up duration, respectively, added as covariates, showed similar results to the main analysis (model fit did not improve in either model with added covariates, Additional file 1: Figure S4).

### Beta-blocker therapy effect on FEV1 in patients with COPD with and without explicit CVD

Data from eight trials evaluating six beta-blockers (atenolol, bisoprolol, carvedilol, celiprolol, metoprolol, and propranolol) in 137 patients with COPD and no explicit CVD were evaluated [51–53, 56, 58–61]. No significant difference in FEV1 was detected when comparing each of the active treatments with placebo (Additional file 1: Figure S5A). Additional file 1: Figure S6 shows celiprolol was similarly likely to rank first in terms of increasing FEV1, while the second rank was surprisingly obtained by placebo, then celiprolol. There were four trials investigating six  beta-blockers (carvedilol, bisoprolol, atenolol, propranolol, metoprolol, labetalol) in patients with COPD and CVD [50, 54, 55, 57]. No significant difference in FEV1 was detected when comparing each of the active treatments with placebo (Additional file 1: Figure S5B, Additional file 1: Figure S7).

### Narrative synthesis

#### Mortality

There were 21 observational studies reporting on mortality [24, 25, 27–41, 43, 44, 64] which evaluated the effect of beta-blockers vs. no beta-blocker use, in an overall population of 422,552 patients from at least 11 countries (Additional file 1: Table S2). According to inclusion criteria, 15 studies enrolled patients with COPD and a CVD indication [25, 27–29, 34, 37, 39, 40, 43, 64, 65], while the remaining six [24, 26, 32, 33, 35, 44] did not specify whether those with CVD were specifically excluded; however, all studies had varying percentages of CVD comorbidities. Overall, patient characteristics varied: mean age ranged between 62.8 [24] and 84.6 [44] years old and the proportion of males between and 37% [26] and 100 [44]%. Distribution of comorbidities was mixed, with hypertension being the most widely reported and ranging between 27.5% [33] and 88.3% [37]. Smoking status was reported in seven studies [25, 26, 28, 31, 33, 41, 44] where most patients were recorded as being either current or former smokers, however data was not available consistently. BMI was reported in five studies [24, 28, 29, 41, 44] only, ranging between 20.4 [29] and 29.9 [24]. Follow-up time was also highly variable, ranging from 2 [30] to 112 months [40].

**Fig. 6:**
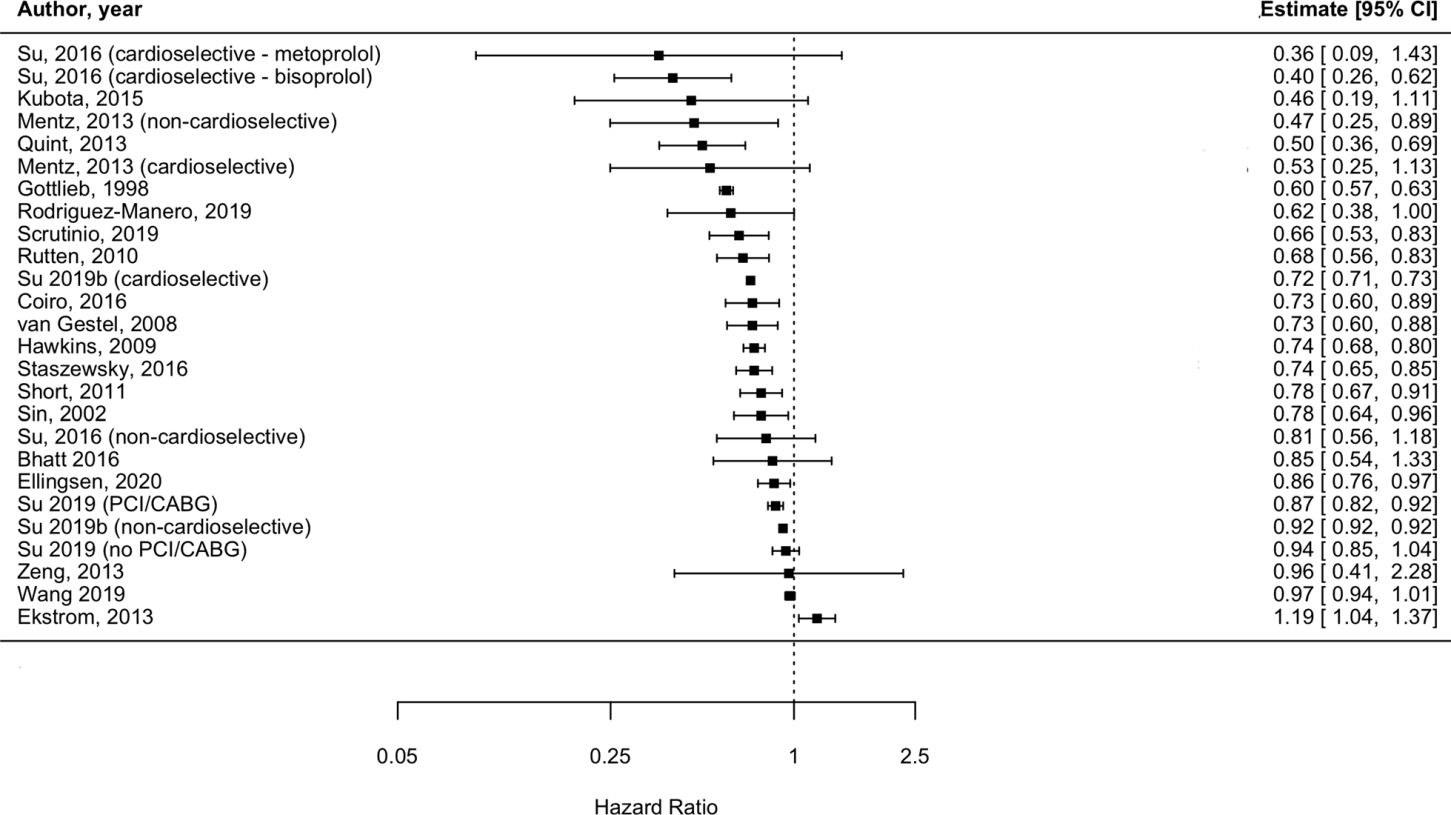
Forest plot illustrating the impact of beta-blocker therapy versus no beta-blocker therapy on mortality, in patients with COPD (Estimate: *HR* hazard ratio, *95% CI* confidence interval)

Individual adjusted study risk estimates for mortality associated with beta-blocker use vs. no beta-blocker use ranged from HR 0.46 (95%CI 0.19–1.11) [29] to 1.19 (95%CI 1.04 to 1.37) [26] (Fig. 6). While age and sex were the most common covariates adjusted for, the majority of studies used a variety of study-specific variables: medications for specific indications (such as hypertension, HF) and other comorbidities or clinical variables (Additional file 1: Table S10). Two studies reported unadjusted analyses [27, 28]. There was one study only reporting an increase in mortality risk associated with beta-blockers (HR: 1.19, 95% CI 1.04 to 1.37); however the population assessed in this report consisted of severe COPD patients who were undergoing long-term oxygen therapy [26]. There was a very high degree of heterogeneity amongst studies (I^2^ = 99.3%). This was explored by conducting stratified analyses (i.e. stratifying by type of beta-blocker [cardioselective vs. non-cardioselective, Additional file 1: Figure S8]; excluding unadjusted estimates; excluding the only study which exclusively included very severe COPD patients). However, due to heterogeneity remaining very high (I^2^ > 75%), results from the outcome analysis are presented graphically (Fig. 6).

#### All-cause hospitalization

All-cause hospitalization was reported in three observational studies [47–49]. One compared cardioselective beta-blockers to non-selective beta-blockers (and presented odds ratios [OR]) [48]; one compared non-cardioselective beta-blockers to selective beta-blockers (and presented HR) [49] and one compared cardioselective beta-blockers to lack of beta-blocker treatment (and presented relative risk) [47] therefore no class-effect comparison could be inferred. None of the studies found significant differences in all-cause hospitalization associated with the investigated treatments (Additional file 1: Table S11).

#### Quality of life

*SGRQ* was assessed in two RCTs [12, 56] and one observational study [45], but none reported mean change from baseline to follow-up per treatment arm. One RCT [66] compared metoprolol to placebo and one observational study [45] assessed any beta-blocker compared to lack of beta-blocker treatment; both reported no significant difference in SGRQ between the two treatment arms at one-year follow-up (Additional file 1: Table S12).

*12MWT* was investigated in two RCTs [59, 62]; one study investigated atenolol and metoprolol vs. placebo, which did not report a mean change in score at  four weeks follow-up [62]; the second study did not find a significant difference in distance walked between patients that received metoprolol vs. propranolol  six hours after treatment was administered [59] (Additional file 1: Table S13).

Data on *6MTW* was reported in two recent RCTs from 2017 [56], respectively 2019 [12]. The first evaluated the effect of bisoprolol compared to carvedilol and did not present mean change between treatment groups, however the calculated estimates suggest both agents decreased distance walked in patients with COPD with no difference apparent between the two; the second trial [12] did not identify a significant difference between the metoprolol and placebo on 6MWT (Additional file 1: Table S14).

Data on *SF-36* was available in one observational study [42]. Whilst overall scores were not available per treatment group, authors reported no significant association between beta-blocker treatment and individual domains of the quality of life assessment tool, either at baseline or 6.4 years follow-up (Additional file 1: Table S15).

### Risk of bias

Observational studies were mostly judged to have moderate risk of bias (23 studies [24–26, 28, 30–35, 37–42, 46–49, 64, 65]), two studies [25, 45] were considered to be of low risk of bias, one [44] had serious risk of bias and one [27] did not provide enough information for a judgment to be made. The domains of bias which were mostly affected by a “moderate rating” were “bias due to confounding” and “bias in selection of participants into study” as the majority of studies included patients recruited from databases which did not provide clinical diagnoses and relied on ICD coding (without confirming validity of diagnosis) (Additional file 1: Table S16). Ten RCTs [53, 55, 58, 60, 61] had moderate risk of bias, denoted by ratings of “some concerns”; two[56, 57] studies were deemed of serious risk of bias, both due to the lack of blinding (Additional file 1: Figure S9).

## Discussion

This comprehensive and up-to-date evaluation of the effects of beta-blockers in patients with COPD adds to the previous literature in several ways: we included all studies reporting on any type of beta-blocker treatment in patients with COPD, showing overall beneficial effects on AECOPD and mortality. For the first time, we used a probabilistic approach to evaluate the effect of beta-blockers on FEV1 using direct and indirect evidence from RCTs in an NMA, comparing seven treatments against placebo, and presented results for patients with COPD with and without CVD disease separately. No beta-blocker affected lung function significantly except propranolol, and the treatments less likely to have a detrimental effect on FEV1 were labetalol (in those with COPD and CVD) and celiprolol (in those with COPD without explicit CVD). Lastly, we found that data on all-cause hospitalization and quality of life endpoints such as SGRQ, 12 and 6MWT and SF-36 were scarcely reported across the literature and did not lend themselves to formal quantitative analysis—suggesting an area of focus for future studies.

### Mortality

Despite heterogeneous elements such as follow-up time, baseline characteristics including age, sex and comorbidities and geographical location, individual results from the 17 out of 21 studies reporting on mortality suggested beta-blocker therapy was associated with a diminished risk of death compared to those not prescribed beta-blockers, in patients with COPD. However, this quality of evidence was deemed “low” per GRADE assessment (Additional file 1: Table S17) and we were not able to quantify the effect of beta-blockers on mortality due to considerable heterogeneity (I^2^ > 75%). Previous reports [10, 11, 67] have provided pooled estimates of reductions in mortality risk associated with beta-blocker treatment, however all reported degrees of heterogeneity above the Cochrane I^2^ threshold of 75%; 89.3% [10], 83% [11] and most recently 96% [67] bringing into question the validity and interpretability of these results as applied to the general COPD population. Reasons for very high heterogeneity in previous meta-analyses include: differences in study populations (i.e. including patients with differing degrees of severity), inaccurate risk of bias assessment and inclusion of different comparators for the intervention effect of interest (i.e. including studies where comparator arms received calcium channel blockers, despite aiming to assess the effect of beta-blocker treatment vs. lack of treatment) [67].

In our analysis, most studies were affected by bias, particularly due to confounding: two studies did not adjust for any covariate factors [27, 55], whilst nine did not adjust for COPD severity either directly, or indirectly by including COPD medication regimen/exacerbation history in the final model [25–28, 30, 32, 34, 36, 37]. Therefore, these studies may overestimate the prognostic effect of beta-blocker therapy on patients with COPD and may, in turn, skew results to show benefits. One of the reasons for the lack of adjustment for COPD-related variables may be due to using data from either existing drug-trials or CVD-specific registries which included data on subgroups of patients with COPD, reiterating the need for trials designed specifically for patients with COPD (with and without additional CVD) which may allow for reliable assessment of the true effect of beta-blockers in these patients. Furthermore, it is not surprising to observe a decrease in mortality, as this could be related to the effect of beta-blockers on other comorbid conditions of patients (i.e. CVD), which is established. A previous study [33] suggested long-term treatment with beta-blockers improved survival of patients with COPD without CVD, however future studies are needed to confirm this result and to assess whether beta-blockers provide non-CV mortality benefits.

### AECOPD

We found evidence to suggest that patients with COPD who are given beta-blockers are at decreased risk of AECOPD (HR 0.78 [95%CI 0.74–0.82]), replicating findings from Du and colleagues [10] who report an even larger reduction in risk, of 37% (RR 0.63 [95% CI, 0.57–0.71]). However, this previous meta-analysis, had methodological limitations inherent to the observational nature of the pooled studies (i.e. residual confounding, immortal time bias), which may limit generalizability of results. However, the GRADE assessment revealed the body of observational evidence on which our estimate was derived was of “low” quality (Additional file 1: Table S19). A recent RCT [12], less likely to be affected by the biases of previous observational studies, found no significant difference between metoprolol and placebo on the time to AECOPD of any severity, but revealed a significant increase in risk of AECOPD requiring hospitalization, in patients with COPD without an indication for beta-blocker treatment, bringing into question the protective effect of this specific beta-blocker agent.

However, this trial did not evaluate other beta-blockers, therefore future RCTs evaluating multiple regimens, are needed to confirm the benefit of these agents. Whether beta-blockers have an indirect effect on exacerbations of COPD could be assessed in clinical trials including patients with COPD and comorbid CVD, allowing assessment of these agents in a more representative COPD population.

### FEV1

FEV1 was assessed in 199 patients enrolled in 12 RCTs and we found that none of the individual cardioselective beta-blockers included in our NMA (atenolol, bisoprolol, celiprolol, metoprolol) were associated with significant effects on lung function in patients with COPD, regardless of baseline FEV1 or follow-up time. This is in line with a Cochrane review [9] which concluded that cardioselective beta-blockers given in either single dose or for longer durations, do not affect FEV1 in patients with COPD, even in those with the lowest baseline FEV1 measurements. Furthermore, our report extends to incorporate a lack of effect on FEV1 of non-selective beta-blockers such as carvedilol and labetalol. Propranolol was the only medication found to be associated with a reduction of 140 ml in FEV1 (95% CrI: -0.28, -0.016), which is larger than the threshold of 100 ml change deemed clinically significant by the American Thoracic Society and European Respiratory Society guidelines. This result is based on high quality evidence, according to the GRADE assessment (Additional file 1: Table S19), and thus supports current recommendations to not use this medication in patients with COPD.

For the first time reported in the literature, we aimed to rank beta-blockers with respect to their effect on lung function. Propranolol had the lowest probability of being ranked first (suggesting worse impact on lung function), compared to all other individual treatments considered in our NMA, including placebo. Labetalol and celiprolol—drugs used in hypertension—were the least likely drugs to negatively impact FEV1, compared to all other beta-blockers; however, neither affected FEV1 with certainty compared to placebo and results were  inferred from very low quality evidence according to GRADE (Additional file 1: Table S18), bringing into question their leading positions in the hierarchy. Since choice of beta-blocker may be influenced by CVD comorbidity (i.e. carvedilol, metoprolol and bisoprolol are recommended in stable HF; atenolol is more often prescribed in patients with asymptomatic hypertension, while bisoprolol is also used in atrial fibrillation, and propranolol is infrequently used to treat tachyarrhythmias), it is perhaps not surprising that we did not identify a clear “best” beta-blocker to be used in COPD. The fact that the beta-blockers less likely to decrease lung function are mainly used to treat hypertension may just reflect this subgroup of patients could be less prone to detrimental side-effects (i.e. indication bias), compared to others with COPD and more severe comorbidities. Indeed, the prescription of beta-blockers in COPD needs to consider clinically significant lung function alteration vs. mortality benefits in those with CVD, particularly MI [68] and HF [69].

Whilst CVD is diagnosed in 20 to 60% patients with COPD [70], our main analysis included primarily small trials and only three explicitly included patients with a cardiac comorbidity (one included angina [54], two included HF patients [55, 57], and one included patients with hypertension, which is a common CVD risk factor [50]. In line with previous research [9], we report no significant FEV1 treatment effect in patients with COPD with CVD.

The remaining eight trials excluded those with CVD (or simply did not report whether this was present), and results mirrored those observed for patients with CVD. Whilst results from this subgroup analysis are encouraging, previous clinical data on in this subgroup is scarce. A recent single RCT including COPD patients without an indication for beta-blockers (therefore those with HF, previous MI or revascularization) failed to demonstrate clear benefits of metoprolol over placebo. Observational studies have included a more varied breadth of specific beta-blockers, however they do not present a clear picture: the population-based Rotterdam Study [71] reported significant decreases in FEV1 associated with both cardio and non-cardioselective beta-blockers, while two other studies, one from Scotland [35] and an one from Japan [72] reported no significant difference in FEV1. Yet, these results may be affected by confounding by indication, which may explain the variability of estimates. Additionally, the longer follow-up times in these studies (ranging from 4 to 6 years) may overlook effects of FEV1 decline which is documented in patients with COPD, regardless of CVD comorbidities.

Overall, our FEV1 analysis suggests the beta-blockers included in this review do not affect lung function in patients with COPD regardless of CVD disease status, and selectivity of agent does not appear to have an impact. However, the two treatment networks contained different medications (celiprolol was assessed in one trial excluding CVD, while labetalol in one trial including CVD) thus we cannot rule out any other potential differential results if a whole range of beta-blockers were included. Finally, we included evidence based on a relatively small population and some of the studies were conducted decades ago; therefore, large clinical studies are needed to assess other agents which may confer lung function benefits across contemporary COPD patients.

The effect of beta-blocker exposure on all-cause hospitalization and quality of life outcomes in patients with COPD could not be quantified, due to a paucity of data. Narrative results from the assessment of studies investigating quality of life outcomes, such as SGRQ, 12 and 6 MWT and SF-36 all suggest non-significant effect of beta-blockers, from both RCTs and observational studies, albeit the data was deemed to be of “very low” quality according to GRADE (Additional file 1: Table S17). Currently, COPD management is focused on preventing exacerbations and improving functioning and health-related quality of life. Clinical studies of beta-blocker treatment in cardiac disease suggests improvements in exercise tolerance and functional status, so whether beta-blockers impair or improve these outcomes in patients with COPD also, is a topic of importance for clinical management. Both randomized trials and, importantly, prospective observational studies with longer follow-up times are needed.

### Limitations

There are several limitations to our analysis: first, we included published, peer-reviewed literature only thus, results may affected by publication bias as it is more likely that studies reporting positive results (i.e. that did not find beta-blockers were associated with negative outcomes) are more often reported than negative studies. Nevertheless, our data is based on the most recent available evidence and portray a nuanced implication of specific beta-blocker treatment in patients with COPD, emphasizing the need for a targeted treatment of CVD comorbidity in these patients.

We only included stable COPD patients and whilst we showed that FEV1 reduction (or increase) was not significant according to beta-blocker exposure (apart for propranolol), we could not verify whether these therapeutic agents diminish the response to rescue COPD medication such as beta-agonists, administered during an exacerbation of COPD. We also did not verify long-term effects of co-administration of beta-blockers and beta-agonists and how their interaction may affect outcomes in patients receiving both types of medication.

Another issue is undiagnosed CVD in patients with COPD. Symptoms of ischemic heart disease or HF may be misattributed or overlapping with COPD, and thus not formally diagnosed, posing difficulties in disentangling possible non-cardiac effects of beta-blockers, independent of their proven cardiac benefits. One advantage of our FEV1 analysis is that we included RCTs only, where concomitant CVD is often ascertained more rigorously and therefore CVD status was known with a greater degree of confidence that may be the case in observational studies.

Furthermore, no statistically significant effect was detected in subgroup analyses stratified by CVD status, which may be due to limited sample size. Future, adequately powered RCTs are needed to assess the effect of beta-blockers in a diverse COPD population, allowing for accurate comparisons based on CVD status to be made.

A recent RCT [12] comparing metoprolol with placebo failed to find a significant effect on FEV1, but reported worsening of dyspnea and overall COPD symptoms, suggestive of respiratory effects not captured by spirometry. This confirms the need to evaluate a spectrum of respiratory outcomes to fully assess the implications of beta-blocker treatment in patients with COPD, which needs to be addressed in future studies

Confounding by contraindication is likely to affect interpretation of results—if we assume clinicians knowingly withheld treatment from patients due to concerns regarding breathlessness, this may have resulted in a reduced sample size of possible COPD patients who may have been eligible for beta-blocker therapy. Alternatively, doctors may prescribe beta-blockers to less severe patients, limiting generalizability.

Our AECOPD analysis is also limited by a low number of included studies, all of which were observational—we identified one RCT only (evaluating metoprolol). This reinstates the need of more carefully conducted RCTs to evaluate a range of beta-blockers and their effects of AECOPD, in order to validate observational data.

## Conclusion

Findings from this analysis represent the most comprehensive and up-to-date available evidence synthesis to assess the effects of beta-blocker use in patients with COPD, spanning data published over four decades. A reduction in COPD exacerbation risk was inferred from observational data while clinical data were pooled to assess lung function. Mortality and quality of life were narratively described owing to high heterogeneity or sparsity of data, respectively. FEV1 was significantly impacted by propranolol, but not by atenolol, bisoprolol, carvedilol, celiprolol, labetalol or metoprolol. In the subset of individuals with CVD, no individual beta-blocker was associated with a reduction in lung function. Treatment choice in patients with COPD should be made according to CVD comorbidity guidelines on management.

## Supplementary Information

**Additional file 1: Figure S1.** Forest plot illustrating results of the meta-analysis evaluating the impact of beta-blocker therapy vs. no beta-blocker therapy on AECOPD in patients with COPD. **Figure S2**. Consistency results illustrating no significant difference between direct and indirect evidence across all comparisons that were assessed in the FEV1 network meta-analysis. **Figure S3**. Comparison-adjusted funnel plot**. Figure S4.** Network meta-analysis with meta-regression results (long vs. short follow-up)*.* **Figure S5**. Network meta-analysis results for patients without A) COPD with explicit cardiovascular disease; B) with cardiovascular disease. **Figure S6.** Rankogram illustrating probabilities of being 1st, 2nd, 3rd…7th with respect to improvement in lung function, for each beta-blocker (and placebo) in patients with COPD without explicit cardiovascular disease. **Figure S7**. Rankogram illustrating probabilities of being 1st, 2nd, 3rd…7th with respect to improvement in lung function for each beta-blocker (and placebo) in patients with COPD with cardiovascular disease. **Figure S8.** Forest plot showing hazard ratios associated with A) Cardioselective beta-blockers and B) Non-cardioselective beta-blockers and mortality in patients with COPD**. Figure S9.** Risk of bias assessment, RCTs. **Table S1**. Screening criteria. **Table S2**. Summary of observational studies. **Table S3.** Patient characteristics—observational studies. **Table S4**. AECOPD estimates for beta-blocker versus no beta-blocker use, from individual observational studies. **Table S5**. Study characteristics—RCTs. **Table S6**. Baseline  characteristics—RCTs. **Table S7**. FEV1 measurements—RCTs. **Table S8**. Network meta-analysis results—league table. **Table S9**. SUCRA ranking probability of being the best treatment. **Table S10.** Mortality estimates for beta-blocker versus no beta-blocker use, from individual studies. **Table S11**. All-cause hospitalization results. **Table S12.** SGRQ results. **Table S13**.12MWT results. **Table S14.** 6MWT results. **Table S15**. SF-36 results. **Table S16**. Risk of bias assessment, observational studies. **Table S17.** GRADE assessment (mortality, quality of life). **Table S18**. GRADE assessment (AECOPD). **Table S19.** GRADE assessment from each pair-wise comparison within the NMA network (FEV1 analysis)

## Data Availability

The datasets analyzed during this study are available from the corresponding author upon reasonable request.

## Abbreviations

6MWT: 6-Minute walking test
12MWT: 12-Minute walking test
AECOPD: Acute exacerbation due to COPD
BMI: Body mass index
COPD: Chronic obstructive pulmonary disease
CI: Confidence interval
CrI: Credible interval
CVD: Cardiovascular disease
FEV1: Forced expiratory volume in one second
HF: Heart failure
HR: Hazard ratio
OR: Odds ratio
NMA: Network meta-analysis
MI: Myocardial infarction
SF-36: Short-Form Health Survey Questionnaire
SGRQ: St. George’s Respiratory Questionnaire
SUCRA: Surface under the cumulative ranking
RCT: Randomized controlled trial
