# Supplementary material for: Beta-blocker therapy in patients with COPD: a systematic literature review and meta-analysis with multiple treatment comparison
Source: Respir Res. 2021 Feb 23;22:64. doi: 10.1186/s12931-021-01661-8 (PMC7903749; doi:10.1186/s12931-021-01661-8)
Supplement: Supplementary file 1 — Additional file 1: Figure S1. Forest plot illustrating results of the meta-analysis evaluating the impact of beta-blocker therapy vs. no beta-blocker therapy on AECOPD in patients with COPD. Figure S2. Consistency results illustrating no significant difference between direct and indirect evidence across all comparisons that were assessed in the FEV1 network meta-analysis. Figure S3. Comparison-adjusted funnel plot. Figure S4. Network meta-analysis with meta-regression results (long vs. short follow-up). Figure S5. Network meta-analysis results for patients without A) COPD with explicit cardiovascular disease; B) with cardiovascular disease. Figure S6. Rankogram illustrating probabilities of being 1st, 2nd, 3rd…7th with respect to improvement in lung function, for each beta-blocker (and placebo) in patients with COPD without explicit cardiovascular disease. Figure S7. Rankogram illustrating probabilities of being 1st, 2nd, 3rd…7th with respect to improvement in lung function for each beta-blocker (and placebo) in patients with COPD with cardiovascular disease. Figure S8. Forest plot showing hazard ratios associated with A) Cardioselective beta-blockers and B) Non-cardioselective beta-blockers and mortality in patients with COPD. Figure S9. Risk of bias assessment, RCTs. Table S1. Screening criteria. Table S2. Summary of observational studies. Table S3. Patient characteristics—observational studies. Table S4. AECOPD estimates for beta-blocker versus no beta-blocker use, from individual observational studies. Table S5. Study characteristics—RCTs. Table S6. Baseline characteristics—RCTs. Table S7. FEV1 measurements—RCTs. Table S8. Network meta-analysis results—league table. Table S9. SUCRA ranking probability of being the best treatment. Table S10. Mortality estimates for beta-blocker versus no beta-blocker use, from individual studies. Table S11. All-cause hospitalization results. Table S12. SGRQ results. Table S13.12MWT results. Table S14. 6MWT results. Table S15. SF- [file 12931_2021_1661_MOESM1_ESM.docx]

**Supplmental Appendix**


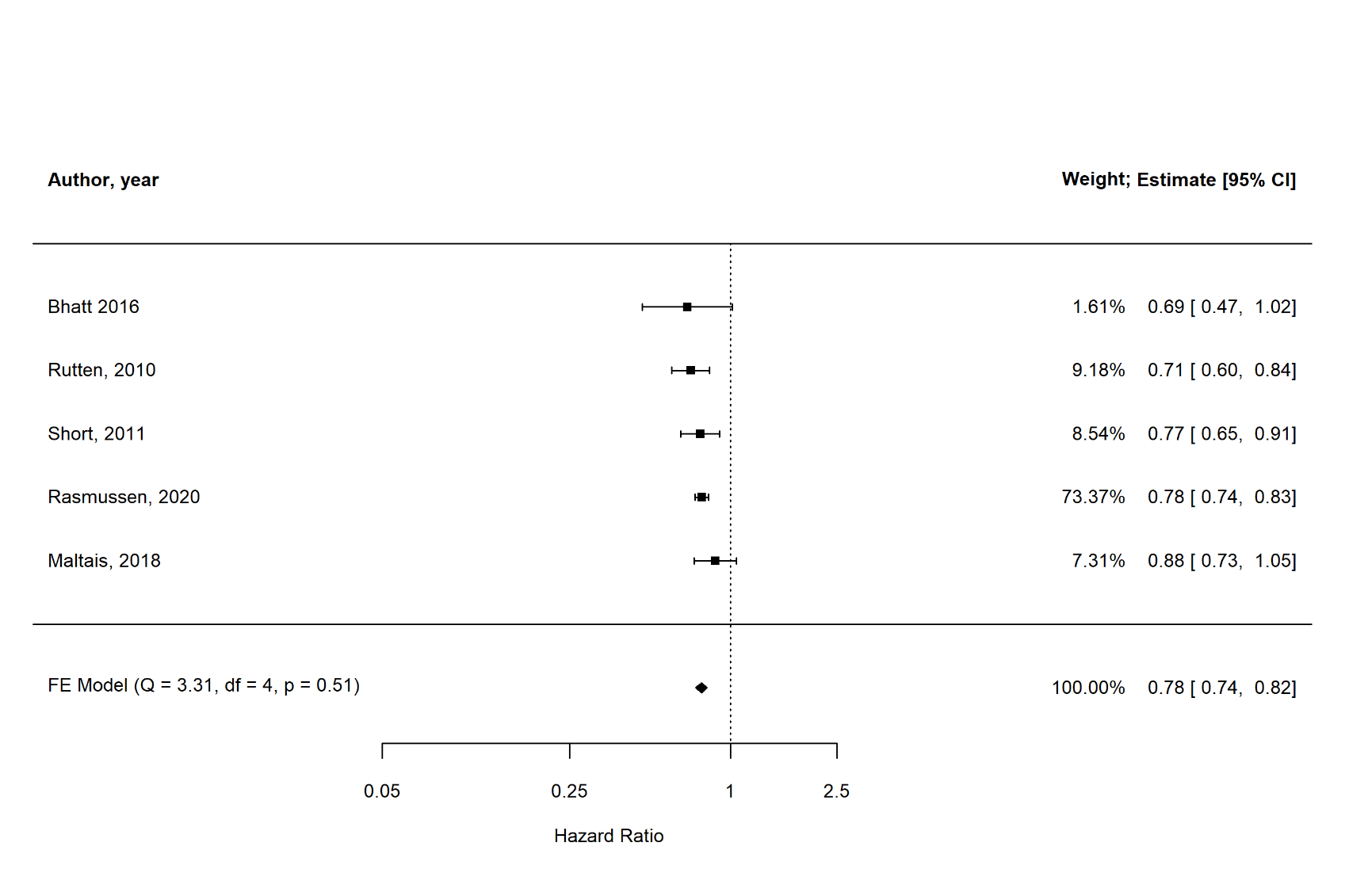


**Figure S1**. Forest plot illustrating results of the meta-analysis evaluating the impact of beta-blocker therapy versus no beta-blocker therapy on AECOPD in patients with COPD, fixed-effects model (Estimate: HR [hazard ratio], 95% CI [confidence interval])


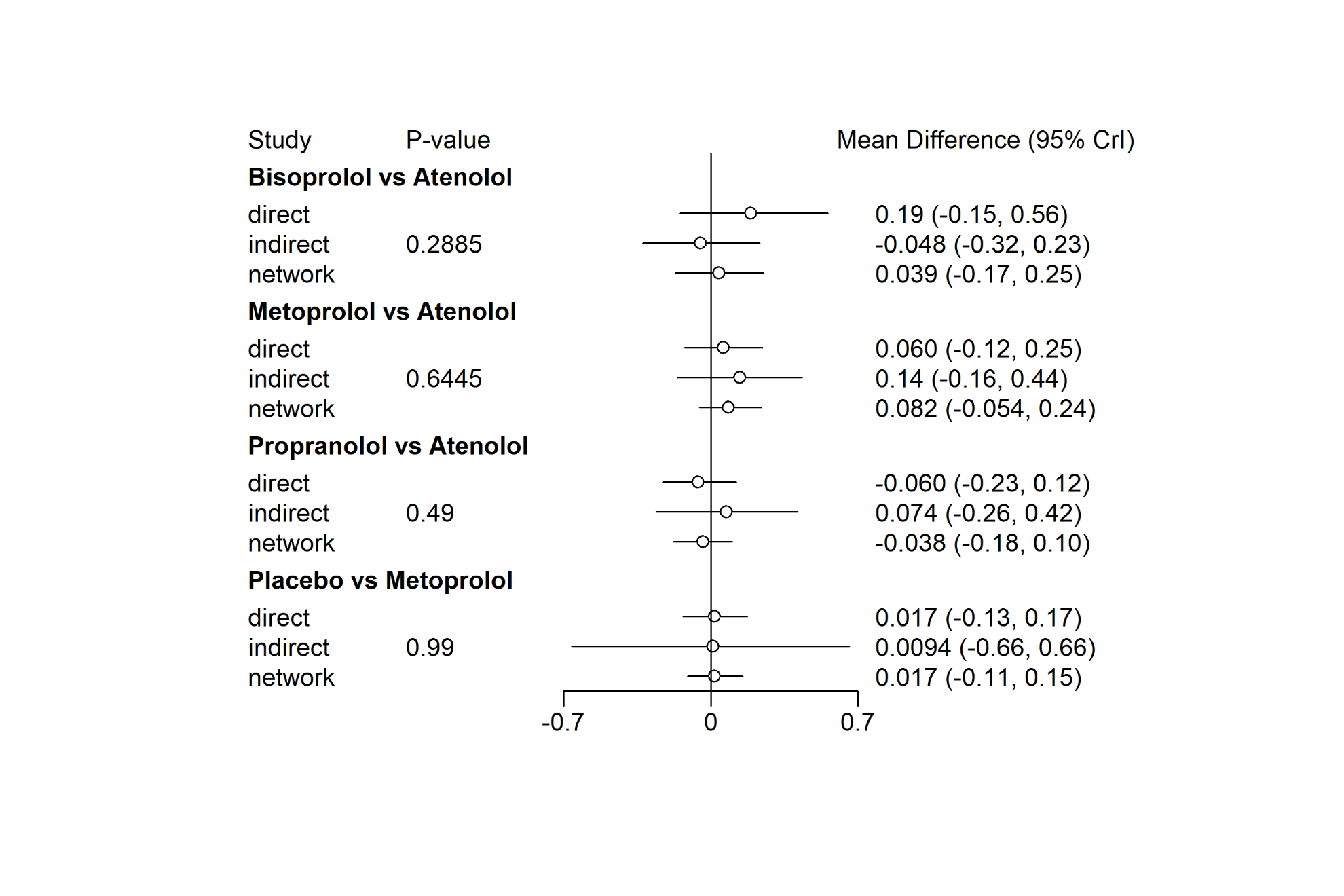


**Figure S2.** Consistency results illustrating no significant difference between direct and indirect evidence across all comparisons that were assessed in the FEV1 network meta-analysis


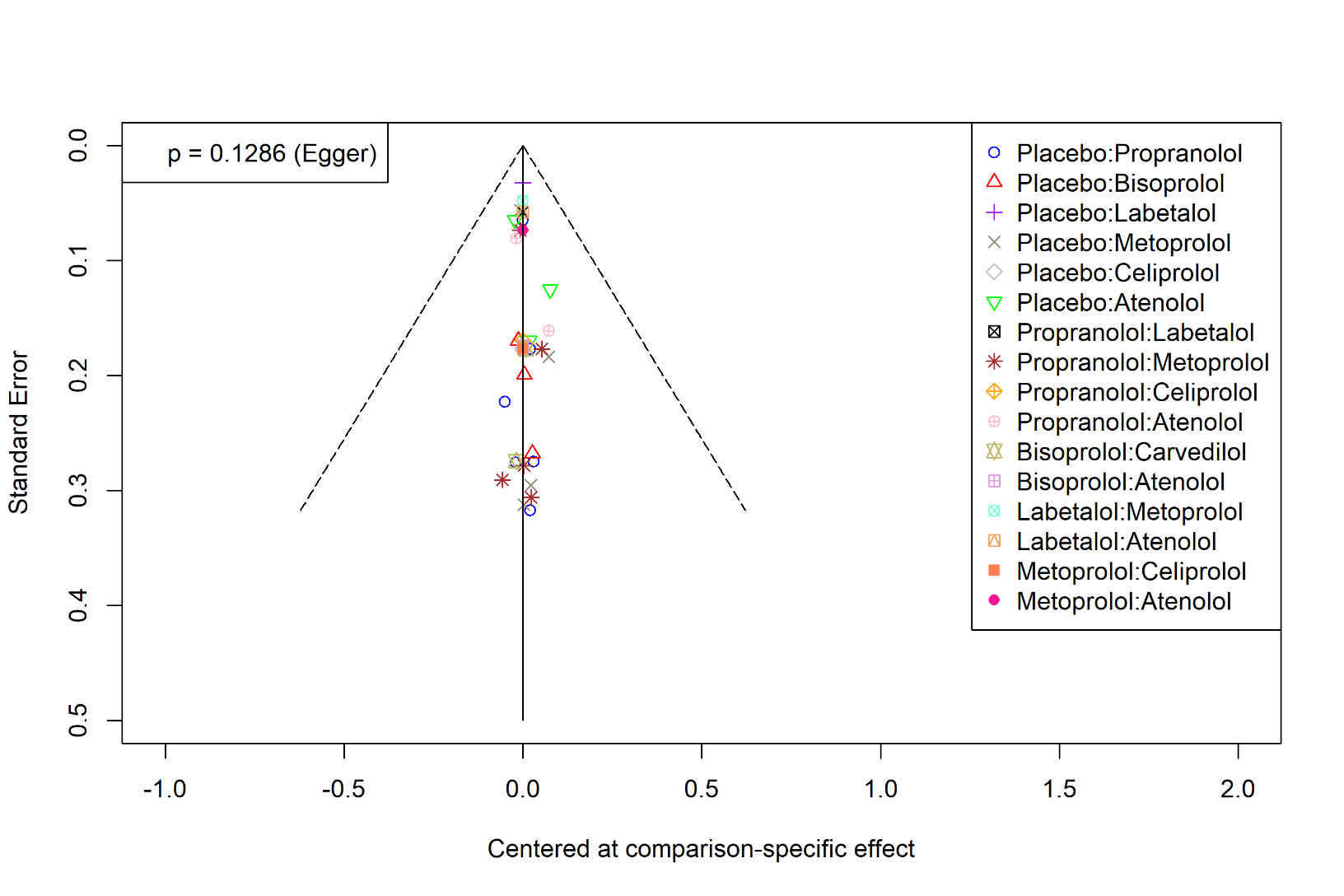


***Figure S3.*** *Comparison-adjusted funnel plot. The vertical line represents the null-hypothesis that the study-specific effect sizes don’t differ from the comparison-specific pooled estimates. The two dashed lines represent 95%CI for the difference between study specific estimates and comparison-specific estimates. The p-value (=0.1286) indicates no significant publication bias.*


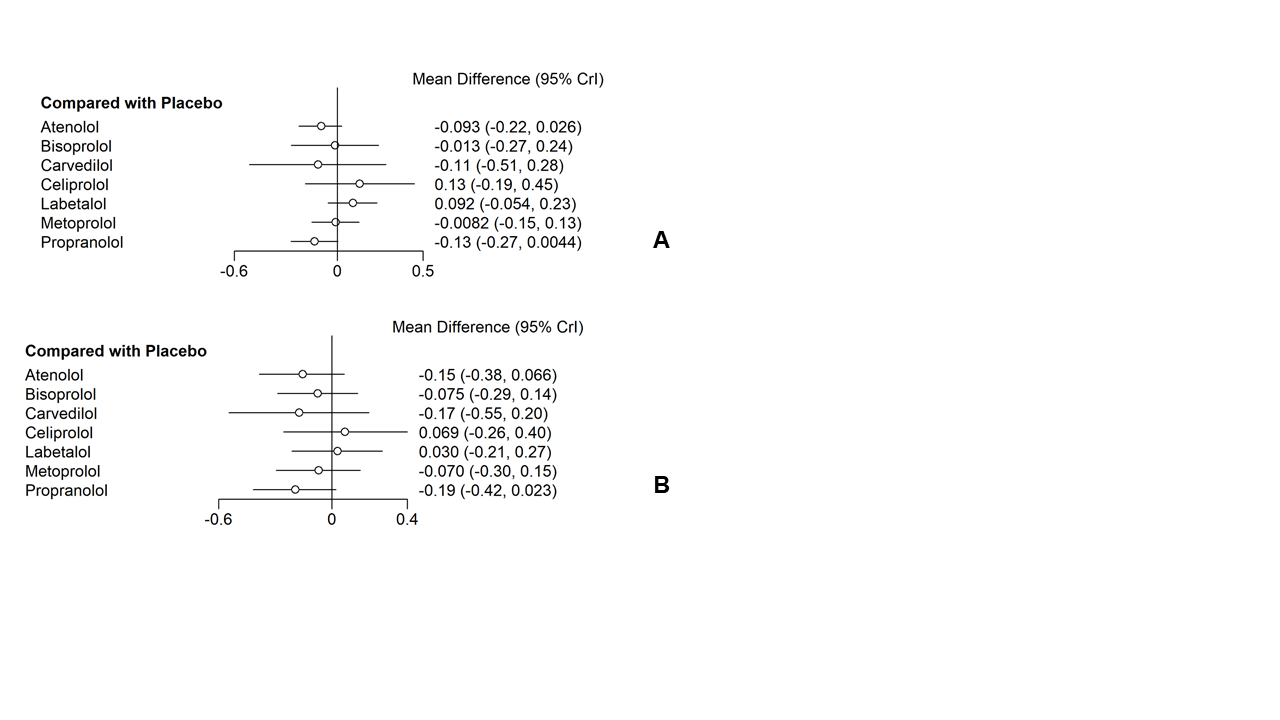


**Figure S4.** Network meta-analysis with meta-regression results (studies with long follow-up, short follow-up)


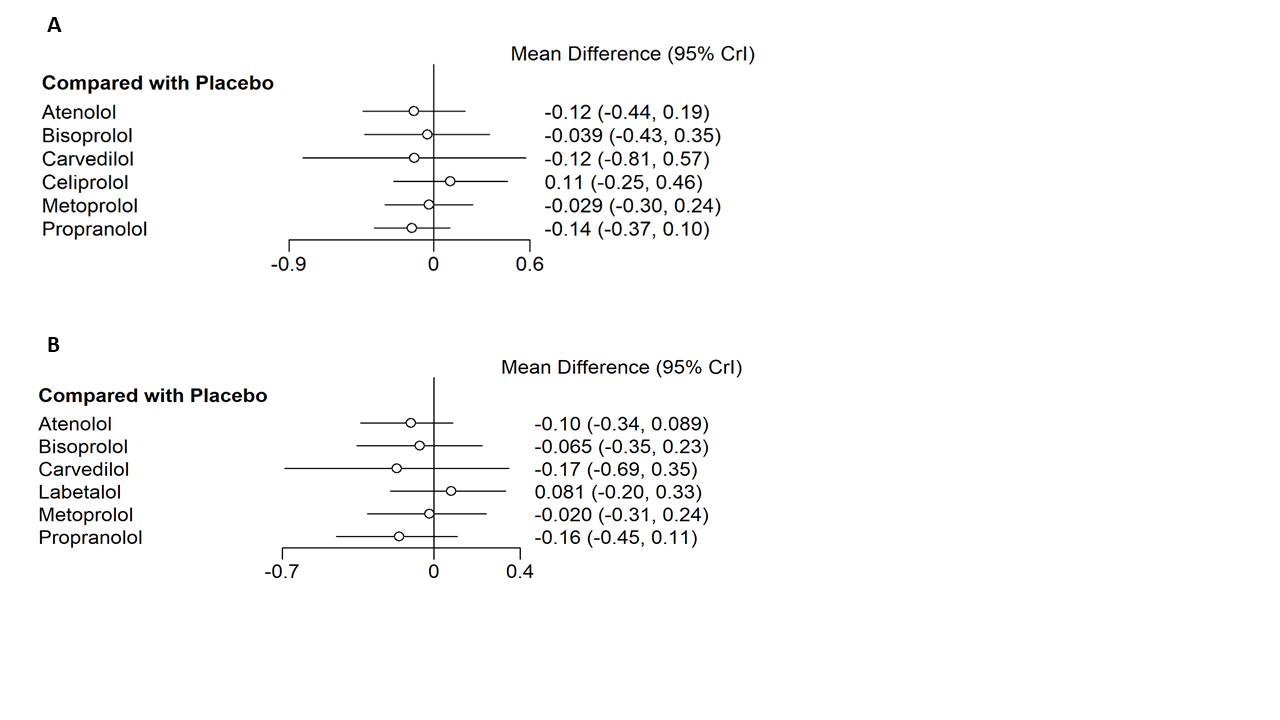


**Figure S5.** Network meta-analysis results for patients with A) COPD without explicit cardiovascular disease; B) with cardiovascular disease; CI=confidence intervals


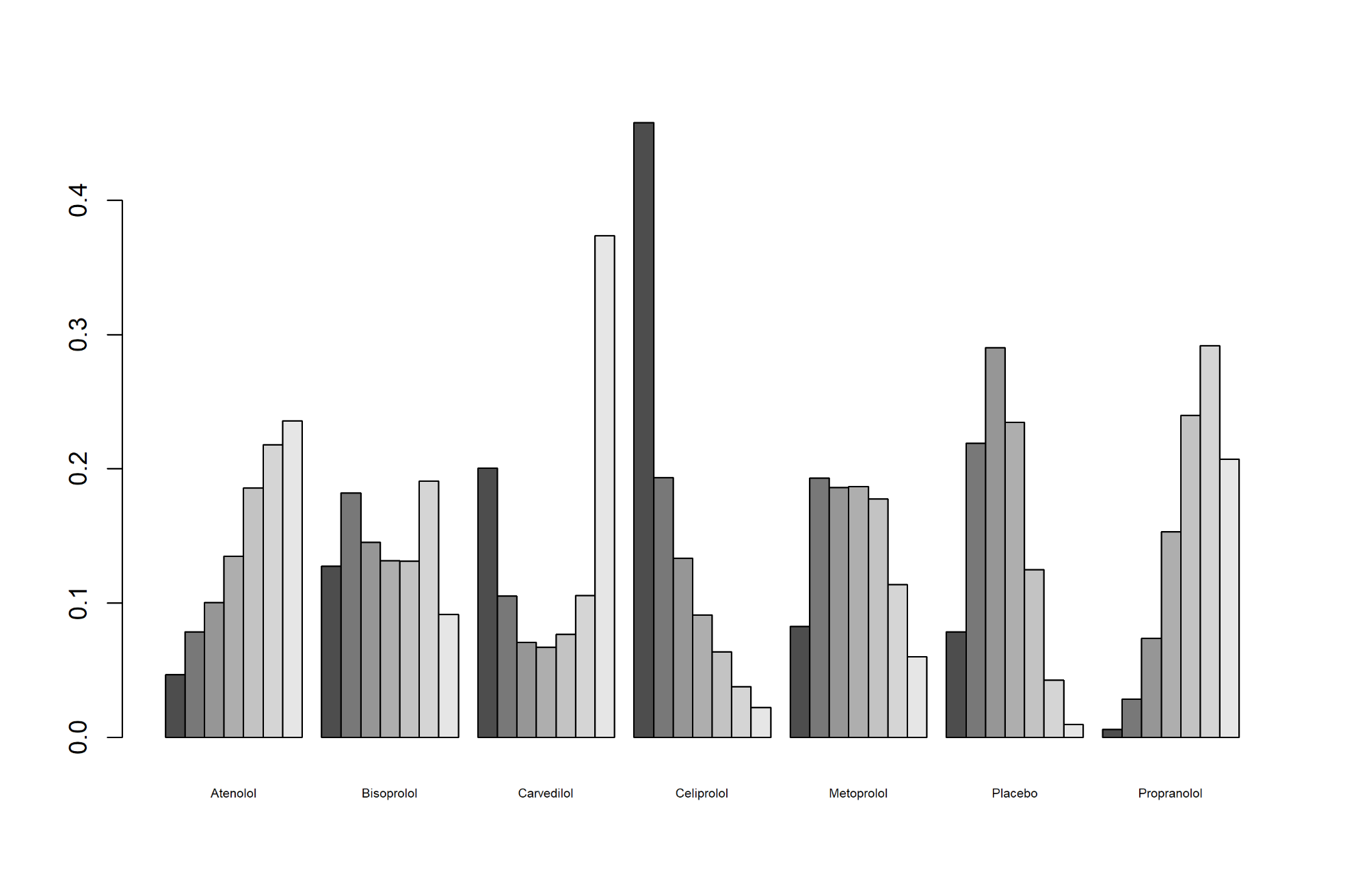


**Figure S6.** Rankogram illustrating probabilities of being 1^st^, 2^nd^, 3^rd^….7^th^ with respect to improvement in lung function, for each beta-blocker (and placebo) in patients with COPD without explicit cardiovascular disease


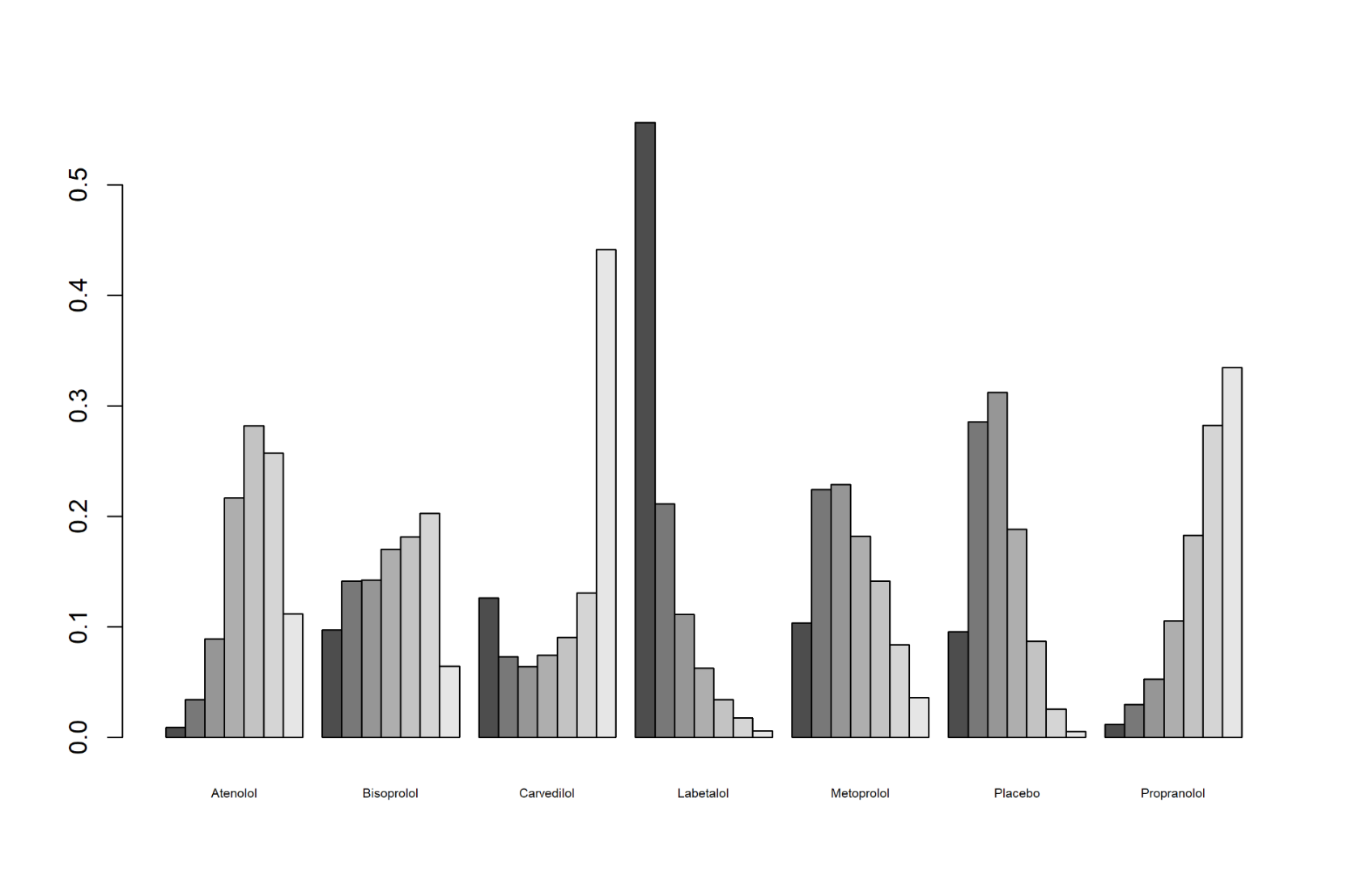


**Figure S7.** Rankogram illustrating probabilities of being 1^st^, 2^nd^, 3^rd^….7^th^ with respect to improvement in lung function for each beta-blocker (and placebo) in patients with COPD with cardiovascular disease


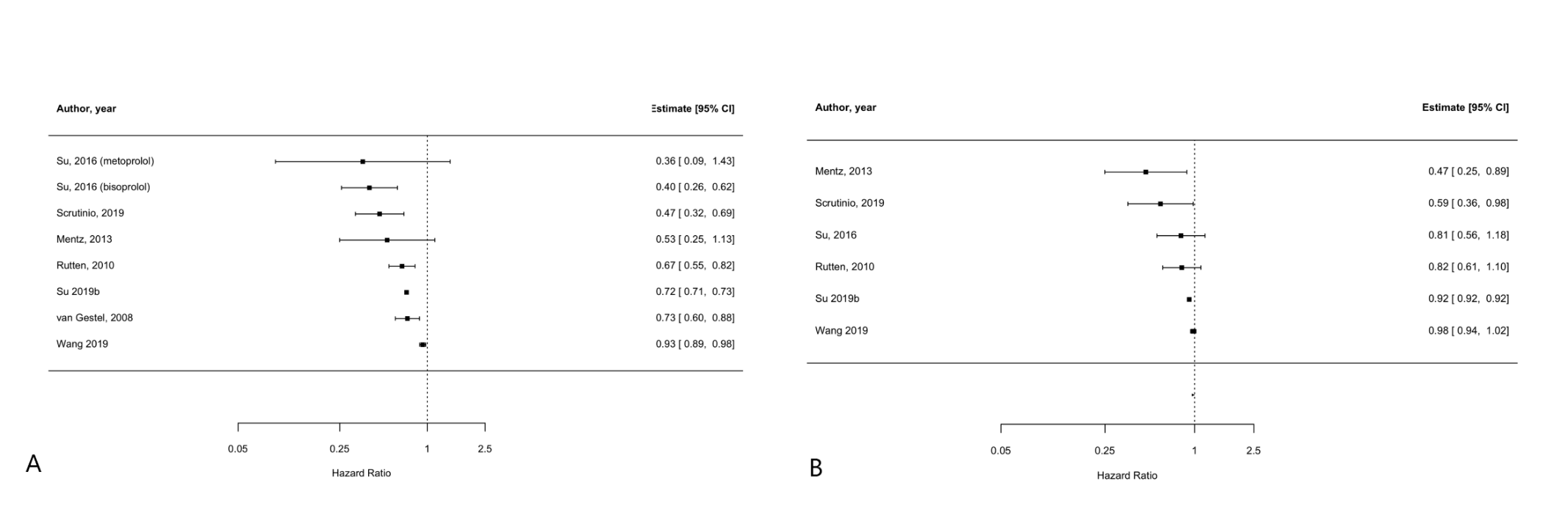


**Figure S8**. Forest plot showing hazard ratios associated with A) Cardioselective beta-blockers and B) Non-cardioselective beta-blockers and mortality in patients with COPD


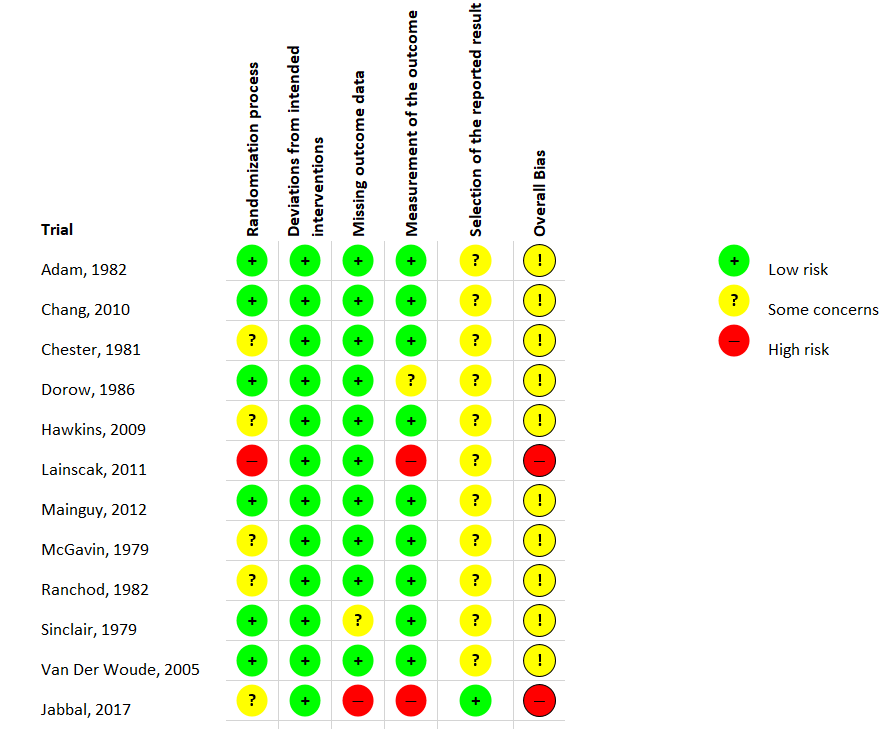


**Figure S9**. Risk of bias assessment, RCTs

| **Category** | **Inclusion Criteria** | **Exclusion Criteria** | **Exclusion reason** |
| --- | --- | --- | --- |
| Population | Adult patients with COPD (Defined as post-bronchodilator FEV1/FVC of <0.70, or as being in accordance with current or previous GOLD guidelines; patients with a clinical diagnosis of COPD) | Publications that do not report data specific to adults with COPD | Population not of interest |
| Interventions | Beta-blockers | Publications that do not report data specific to beta-blockers | No treatment of interest |
| Comparators | Placebo or any beta-blocker (above) | Studies reporting on treatments other than beta-blockers | No comparison of interest |
| Outcomes | Mortality, AECOPD, FEV1, all-cause hospitalization, quality of life (SGRQ, 12 and 6MWT, SF-36) | Publications that do not report clinical efficacy or safety data on potential comparators | Relevant outcomes not reported |
| Study design | Observational studies, RCTs | - Animal studies - In-vitro/ex-vivo studies - Case studies/case series - Reviews, editorials, conference abstracts (we will include only peer-reviewed publications) | Study design not of interest |
| Language | English-language articles only | Journal articles with non-English full-texts | Non-English |
| Geographical regions | No limitation based on geographic region(s) | N/A | N/A |
| Publication date | Studies published until January 2021 | N/A | N/A |
| COPD, chronic obstructive lung disease; FEV1, forced expiratory volume in 1 second; FVC, forced vital capacity; GOLD, Global Initiative for Chronic Obstructive Lung Disease; RCT, randomized controlled trial; N/A, not applicable | | | |

***Table S1.*** *Screening criteria*

**Table S2.** Summary of observational studies included in the systematic literature review

| **Author, country** | **Beta-blocker evaluated** | **Outcomes** | **Follow-up** | **Population** | **Sample size** | **Notes** |
| --- | --- | --- | --- | --- | --- | --- |
| Rutten, 2010; Netherlands | Cardioselective; non-cardioselective | All-cause mortality,  AECOPD | Mean 7.2 years (±2.8) | COPD | 2230 | Mortality, Meta-analysis AECOPD |
| Maltais, 2018; Multiple countries | Not specified (any BB) | AECOPD, SGRQ | 1 year | COPD (GOLD stage 2-4) | 5162 | Meta-analysis AECOPD |
| Short, 2011 ; Scotland | Cardioselective (88%); non-cardioselective | All-cause mortality;  AECOPD | Mean 4.35 years (±2.28) | COPD | 5977 | Mortality, Meta-analysis of AECOPD |
| Bhatt 2016; UK | Not specified (any BB) | AECOPD, all-cause mortality, SGRQ | Median 2.1 years | COPD | 3464 | Mortality, Meta-analysis of AECOPD |
| Rasmussen 2020; Denmark | Cardioselective and non-cardioselective (any BB) | AECOPD | Median: 0.76 years | COPD + MI | 10,884 | Meta-analysis of AECOPD |
| van Gestel, 2008 ; Netherlands | Cardioselective | All-cause mortality | Median 5 years | COPD + vascular surgery | 1265 | Mortality |
| Quint, 2013 ; England | Cardioselective;  non-cardioselective | All-cause mortality | Median 2.9 years (range 0.09 - 7.2) | COPD + first MI | 1063 | Mortality |
| Zeng, 2013; China | Cardioselective; non-cardioselective | All-cause mortality | Median 1.85 years | COPD | 220 | Mortality |
| Mentz, 2013; USA | Cardioselective; non-cardioselective | All-cause mortality | 2 months | COPD + HF | 725 | Mortality |
| Gottlieb, 1998; USA | Not specified (any BB) | All-cause mortality | 2 years | COPD + acute MI | 48,480 | Mortality |
| Sin, 2002; Canada | Not specified (any BB) | All-cause mortality | Median: 21 months (IQR, 7 - 39) | COPD + HF | 3834 | Mortality |
| Ekstrom, 2013; Sweden | Cardioselective (98%) | All-cause mortality | Median 1.1 years (IQR 0.6-2) | COPD | 1794 | Mortality |
| Coiro, 2016; Multiple countries | Not specified (any BB) | All-cause mortality, | Mean 2.7 years | COPD + acute MI | 1573 | Mortality |
| Staszewsky, 2016; Italy | Not specified (any BB) | All-cause mortality | 4 years | COPD + HF | 2837 | Mortality |
| Su, 2016; Taiwan | Cardioselective; non-cardioselective | Survival | Mean 4.35 years (±2.28) | COPD + HF | 11,558 | Mortality |
| Kubota, 2015; Japan | Cardioselective; non-cardioselective | All-cause mortality | Mean 2.75 years | COPD + acute HF | 132 | Mortality |
| Hawkins, 2009; Italy | Not specified (any BB) | All-cause mortality | N/R | COPD + history of MI | 1258 | Mortality |
| Ellingsen, 2020; Sweden | Not specified (any BB) | All-cause mortality;  AECOPD | 10 years | COPD + AF | 17,745 | Mortality |
| Rodriguez-Manero, 2019; Spain | Not specified (any BB) | All-cause mortality | Mean 1.93 years (±0.28) | COPD | 937 | Mortality |
| Su, 2019b; Taiwan | Cardioselective; non-cardioselective | All-cause mortality | 9.32 years | COPD + HF | 275,436 | Mortality |
| Su 2019; Taiwan | Cardioselective; non-cardioselective | All-cause mortality | Mean:  BB group: 3.9 years (± 2.7);  control group: 3.5 years (± 2.7) | COPD + acute MI | 22,007 | Mortality |
| Wang 2019; Taiwan | Cardioselective; non-cardioselective | All-cause mortality | N/R | COPD + first acute MI | 23,116 | Mortality |
| Scrutinio, 2019; Italy | Cardioselective; non-cardioselective | All-cause mortality | N/R | COPD + HF | 396 | Mortality |
| Farland, 2013; USA | Cardioselective; non-cardioselective | All- hospitalization | 1 year | COPD | 412 | Outcome not reported in suitable format (OR); only two observational studies per this outcome |
| Brooks, 2007; USA | Cardioselective; non-cardioselective | All-cause hospitalization | N/R | COPD | Not reported per population of interest | Only two observational studies per this outcome |
| van Gestel 2009; Netherlands | Not specified (any BB) | SF-36 | 6.4 years | COPD + PAD | 1310 | Only one observational study reporting on this outcome |
| AECOPD, acute exacerbation of COPD; AF, atrial fibrillation; BB, beta-blocker; DM, diabetes mellitus, GOLD, Global Initiative for Chronic Obstructive Lung Disease; HF, heart failure; MI, myocardial infarction, PAD, peripheral artery disease; SF-36, Short-Form Health Survey Questionnaire; SGRQ, St. George’s Respiratory Questionnaire; USA, United States of America | | | | | | |

**Table S3.** Patient characteristics observational studies

| **Author** | **Group** | **No.**  **patients** | **Age mean (SD), median [IQR]** | **% Males** | **% HTN** | **% DM** | **% MI** | **%IHD** | **% Other comorbidities** | **BMI (SD)** | **Smoking status (%)** |
| --- | --- | --- | --- | --- | --- | --- | --- | --- | --- | --- | --- |
| Rutten, 2010 | BB | 665 | 64.7 (10.3) | 49.8 | 66.8 | 24.1 | 9.6 | 38.3 | HF: 32  AF:18.8  Stroke:9 | - | Current smokers34.6  Non-smokers: 14.4  Former smokers: 51 |
| Rutten, 2011 | No BB | 1565 | 64.8 (10.3) | 54.4 | 27.5 | 13.5 | 2.6 | 11.8 | HF: 21.3  AF: 6.2  Stroke: 6.1 | - | Current smokers: 38.3  Non-smokers : 13.3  Former smokers: 48.3 |
| Maltais, 2018 | BB | 557 | 65.0 (7.7) | 70.6 | 84 | - | 9.9 | 51.9 | - | 28.5 (5.6) | Current smokers: 35.5  Former smokers: 64.5 |
| Maltais, 2018 | No BB | 4605 | 63.9 (8.4) | 73.2 | 38.8 | - | 1.7 | 17.8 | - | 25.5 (5.4) | Current smokers: 37.1  Former smokers: 62.9 |
| Short, 2011 | No BB | 1180 | 70.5 (10.2) | 52.3 | - | 12.3 | - | - | CVD 45.3 | - | - |
| Short, 2011 | BB | - | - | - | - | - | - | - | - | - | - |
| Bhatt 2016 | BB | 474 | 66.8 (7.7) | 60.1 | 84.4 | 24.1 | - | 44.7 | HF:4.1; CVA: 5.2; PAD: 2.8 | 29.9 (6.7) | - |
| Bhatt 2016 | No BB | 2990 | 62.8 (8.5) | 54.9 | 45.4 | 10.9 | - | 11.8 | HF: 12.2; CVA: 10.8; PAD: 7.8 | 27.8 (6.2) | - |
| Rasmussen, 2020 | BB + no BB | 10,884 | 74 [68-81] | 52 | 44.4 | 20.3 | 100 |  | AF: 22.5  HF: 33.2  Angina: 26.1  PVD: 14  CVA: 13.2  Cancer: 14.1  CKD: 7.9 | - | - |
|  |  |  |  |  |  |  |  |  |  |  |  |
| van Gestel, 2008 | BB | 462 | 69 (9) | 82 | 49 | 17 | 33 | - | - | 26 (4) | Current smokers: 35 |
| van Gestel, 2008 | No BB | 803 | 69 (10) | 78 | 36 | 12 | 21 | - | - | 25 (4) | Current smokers : 33 |
| Quint, 2013 | No BB | 586 | - | 60.4 | 47.8 | 6 | - | - | HF: 14.7  PAD: 13.1 | - | Current smokers :35.3  Non-smokers: 5.3  Former smokers: 58.9 |
| Quint, 2013 | BB at MI | 233 | - | 68.2 | 41.6 | 5.2 | 100 | - | HF: 7.3;  PAD: 9.4 | - | Current smokers: 42.9  Non-smokers: 4.3  Former smokers: 52.8 |
| Zeng, 2013 | BB | 81 | 84.6 (6.2) | 100 | 75.3 | 48.1 | - | 86.4 | HF 23.5% | 24.7 (2.6) | Current smokers 30.9 |
| Zeng, 2013 | no BB | 76 | 82.6 (8.5) | 100 | 68.4 | 42.1 | - | 69.2 | HF 17.1% | 24.1 (3) | Current smokers: 38.2 |
| Mentz, 2013 | BB + no BB* | 725 | median 73 (63-80) | 63 | 69 | 43 | - | - | - | - | - |
| Gottlieb, 1998 | BB + no BB* | 48480 | - | - | - | - | - | - | - | - | - |
| Sin, 2002 | BB | 242 | - | - | - | - | - | - | - | - | - |
| Sin, 2002 | no BB | 3592 | - | - | - | - | - | - | - | - | - |
| Ekstrom, 2013 | BB | 829 | 75.6 (7.8) | 44 | - | 19 | - | - | CVD: 1 = 40%, 2 = 30%, >2 = 17% | - | Former smokers: 19.9 |
| Ekstrom, 2013 | no BB | 965 | 74.1 (8.6) | 37 | - | 5 | - | - | CVD: 1 = 33%, 2 = 10%, >2 = 4% | - | Former smokers: 18 |
| Coiro, 2016 | BB | 822 | 66 (10) | 75 | 63 | 31 | 40 | - | Previous MI: 40; AF: 16 | - | Current smokers:16  Non-smokers: 38  Former smokers: 38 |
| Coiro, 2016 | No BB | 751 | 69 (9) | 69 | 58 | 30 | 36 | - | Previous MI: 36; AF: 21 | - | Current smokers: 19  Non-smokers: 41  Former smokers: 41 |
| Staszewsky, 2016 | BB + no BB* | 2837 | 78.8 (8.9) | 60.9 | 88.3 | 29.5 | - | - | CAD: 45.5 | - | - |
| Su, 2016 | BB + no BB* | 11558 | 71 (63–76) | 53.4 | - | 33.6 | - | 62.9 | - | - | - |
| Kubota, 2015 | Carvedilol | 52 | 78.2 (8.2) | 91.6 | 60 | - | - | 44.4 | AF: 42.5% CKD: 68% | 21.2 3.1() | - |
| Kubota, 2015 | Bisoprolol | 34 | 79.1 (6.5) | 89.7 | 66.7 | - | - | 31.8 | AF: 44.1% CKD: 68% | 20.5 (4.6) | - |
| Kubota, 2015 | BB | 86 | 78.5 (6) | 90.9 | 62.3 | - | - | 38.1 | CKD: 64.1  AF: 43.3 | 20.9 (3.8) | - |
| Kubota, 2015 | no BB | 46 | 79.5 (9.2) | 78.6 | 52.6 | - | - | 34.2 | CKD: 50  AF:58.8 | 20.4 (3.3) | - |
| Hawkins, 2009 | BB + no BB* | 1,258 | 68.1 (9.9) | 71.1 | 58.1 | 25.7 | 39.9 | - | - | 27.5 (5.50) | Current smokers:42  Former smokers: 41.2 |
| Ellingsen, 2020 | BB + no BB * | - | - | - | - | - | - | - | - | - | - |
| Rodriguez-Manero, 2019 | BB + no BB * | 937 | 82 (7.9) | 67.9 | 71.1 | 26.8 | - | - | HF: 41.5%  AF: 11.1% Vasculopathy: 8.5%; | - | - |
| Su, 2019b | BB + no BB * | 275436 | 70.63 (9.87) | 59.14 | 81.33 | 37 | - | 51.94 | Malignancy :14.16%  Cirrhosis: 24.74;  CVD: 27.64; CKD:5.25 | - | - |
| Su 2019 | BB | 10638 | 70 (61-78) | 77 | 68 | 37 | 100 | 100 | HF:13;  Stroke: 12; CKD:10;  AF: 4 | - | - |
| Su 2019 | No BB | 11369 | 74 (65-80) | 77 | 65 | 36 | 100 | 100 | HF: 17  Stroke: 15  CKD:11  AF: 5 | - | - |
| Wang 2019 | Cardioselective BB | 2859 | 76.1 (10.06) | 74.92 | 78.31 | 44.07 | 100 | 100 | HF:43  Stroke: 37.6 AF:19.3 | - | - |
| Wang 2019 | Non-cardioselective BB | 4750 | 75.45 (10.02) | 73.24 | 72.11 | 42.08 | 100 | 100 | HF: 42  Stroke: 36.8 AF:18.3 | - | - |
| Wang 2019 | No BB | 15507 | 77.63 (9.61) | 74.02 | 69.13 | 37.32 | 100 | 100 | HF:42.3  Stroke: 37.4  AF: 19.6 | - | - |
| Scrutinio, 2019 | BB + no BB* | 396 | 74 (11) | 75.7 | 64.1 | 34.2 |  | 51.6 | - | - | - |
| Sessa, 2018a | Non-cardioselective | 3902 | 72.6 (9.9) | 66.9 | 59.5 | 30 | 43 | - |  | - | - |
| Sessa, 2018a | Cardioselective | 10437 | - | - | - | - | - | - | - | - | - |
| Sessa, 2018a | BB + no BB | 14449 | 74.7 (9.9) | 58.5 | 64.1 | 29.2 | 40.5 | - | - | - | - |
| Farland, 2013 | BB | 166 | 64 (10.8) | 47.6 | 91.6 | 40.4 | - |  | CVD: 61.4 | 45.8 | Current smokers: 17.5  Non-smokers: 36.7 |
| Farland, 2013 | No BB | 246 | 60.8 (11.8) | 38.6 | 65 | 24.4 | - |  | CVD: 33.7 | 54.1 | Current smokers: 11  Non-smokers: 35 |
| Brooks, 2007 | Cardioselective BB | 3062 | 63.2 (13.7) | - | - | - | - | - | - | - | - |
| Brooks, 2007 | Non-cardioselective BB | 690 | 58.6 (17.4) | - | - | - | - | - | - | - | - |
| Brooks, 2007 | No BB | 7840 | 64.4 (13.4) | - | - | - | - | - | - | - | - |
| van Gestel 2009 | BB | 191 | 68 (9) | 81 | 46 | 20 | 33 | - | - | - | - |
| van Gestel 2009 | No BB | 135 | 64 (11) | 79 | 39 | 4 | 16 | - | - | - | - |
| *Data not available separately  SD, standard deviation; IQR, interquartile range; HTN, hypertension, DM, diabetes mellitus; MI, myocardial infarction; BMI, body mass index; IHD ischemic heart disease; BB, beta-blocker; AF, atrial fibrillation; HF, heart failure; CKD, chronic kidney disease; CVD, cardiovascular disease; CAD, coronary artery disease; No, number | | | | | | | | | | | |

**Table S4.** AECOPD estimates for beta-blocker versus no beta-blocker use, from individual observational astudies

| **Author** | **Comparison** | **Follow-up (months)** | **AECOPD HR [95 CI]** | **Covariates adjusted for in analysis** |
| --- | --- | --- | --- | --- |
| Rutten, 2010 | BB vs. no BB | 86.4 | 0.71 [0.6 – 0.83] | Age, sex, smoker, diabetes, hypertension, CVD, pulmonary drugs, referral to pulmonologist |
| Maltais, 2018 | BB vs. no BB | 12 | 0.88 [0.73 – 1.05] | Age, sex, COPD treatment, BMI, race, GOLD stage, cardiac disorders, hypertension, ACEI, ARB, lipid-modifying agents |
| Short, 2011 | BB vs. no BB | 52 (27) | 0.77 [0.65 -0.91 | History of hospital admission for CVD, diabetes, smoking, age, sex, FEV1, resting Sa02 and deprivation index |
| Bhatt 2016 | BB vs. no BB | median 25.2 | 0.69 [0.47 – 1.02] | Age, race, HF, FEV1, % emphysema on CT, respiratory medications, log CAC and the propensity to prescribe BB |
| Rasmussen, 2020 | BB vs. no BB | Mean 9.1 months | 0.78 [0.74 – 0.83] | Age, sex, history of AECOPD, inhaled therapy, comorbidities, type of MI, revascularization procedures, income, calendar year |
| ACEI, angiotensin-converting-enzyme inhibitors; ARB, angiotensin receptor blockers; AECOPD, acute exacerbation due to COPD; BB, beta-blockers; BMI, body mass index; CAC, coronary artery calcification; CVD, cardiovascular disease; CT, computed tomography; FEV1, forced expiratory volume in 1 second HF, heart failure; GOLD, Global Initiative for Chronic Obstructive Lung Disease; MI, myocardial infarction; Sa02, arterial oxygenation saturation; | | | | |

**Table S5.** Study characteristics (RCTs)

| **Author, year, country** | **Primary Outcome** | **Secondary Outcomes** | **Study design** | **Population** | **Drug** | **Included in NMA** | **Notes** |
| --- | --- | --- | --- | --- | --- | --- | --- |
| Adam 1982, Australia | FEV1, 3.5 h after drug administration | Specific AWR | Double-blind, placebo-controlled cross-over | COPD + HTN | Labetalol, metoprolol, atenolol, propranolol, placebo | Yes | FEV1 |
| Hawkins 2009, UK | FEV1 | Other pulmonary function tests, symptoms and quality of life | Double-blind, placebo controlled | COPD + HFrEF | Bisoprolol, placebo | Yes | FEV1 |
| Lainscak 2011, Slovenia | FEV1 | Pulmonary function, heart rate, NT-proBNP. | Open-label | COPD + HFrEF | Bisoprolol, carvedilol | Yes | FEV1 |
| McGavin 1979, UK | FEV1 | 12 MWT; pulse rate; FVC; PEFR | Double-blind cross-over | COPD | Metoprolol, propranolol | Yes | FEV1 |
| Van Der Woude 2005, Netherlands | FEV1 | Bronchoconstriction | Double-blind, placebo-controlled cross-over | COPD | Metoprolol, celiprolol, propranolol | Yes | FEV1 |
| Mainguy 2012, Canada | Difference in dynamic hyperinflation | Differences in cycle endurance test duration, pulmonary function | Double-blinded cross-over | COPD | Bisoprolol | Yes | FEV1 |
| Chang 2010, New Zealand | FEV1 | Exercise capacity, salbutamol response curve | Double-blind placebo-controlled cross-over | COPD, moderate | Propranolol, metoprolol | Yes | FEV1 |
| Jabbal 2017, UK | AWR | FEV1, FVC, RVC, SGRQ, TDI, 6MWT | Open-label, cross-over | COPD, moderate to severe | Bisoprolol, carvedilol | Yes | FEV1 |
| Trials with SDs extrapolated for NMA analysis | | | | | | | |
| Dorow 1986, Germany | AWR | FEV1 | Double-blind, placebo-controled cross-over | COPD + stable angina | Bisoprolol, atenolol, placebo | Yes | SD extrapolated* |
| Sinclair 1979, UK | FEV1 | Symptoms | Double-blind, placebo-controlled cross-over | COPD | Propranolol, metoprolol | Yes | SD extrapolated* |
| Chester 1981, USA | FEV1 | FVC | Double-blind cross-over | COPD | Propranolol | Yes | SD extrapolated* |
| Ranchod 1982, South Africa | FEV1 | Pulse rate, FEV1, MMFR, PFR | Double-blind placebo-controlled cross-over | COPD | Propranolol, atenolol | Yes | SD extrapolated* |
| Excluded from NMA | | | | | | | |
| Butland 1980, UK | FEV1 | 12MWT | Double-blind placebo-controlled cross-over | Emphysema with  severe airway obstruction | Metoprolol, atenolol, placebo | No | Mean change in FEV1 or data needed to calculate not provided |
| Dransfield 2019, USA | AECOPD | All-cause mortality, all-cause hospitalization, spirometry, 6MWT | Double-blind, placebo-controlled | COPD, moderate to severe | Metoprolol | No | Only one RCT reporting on AECOPD |
| *SD was extrapolated (averaged) from studies with similar characteristics  12MWT, 12 minutes walking test; 6MWT, 6 minutes walking test; AECOPD, acute exacerbation of COPD; AWR, airway resistance; FEV1, forced expiratory volume in 1 second; FVC, forced vital capacity; HFrEF, heart failure with reduced ejection fraction; MMFR, maximal mid-expiratory flow rate; NMA, network meta-analysis; NT-proBNP, N-terminal pro-brain natriuretic peptide; PFR, peak expiratory flow rate; RCT, randomized controlled trial, SD, standard deviation; SE, standard error; SGRQ, St. George’s Respiratory Questionnaire; TDI, transition dyspnea index | | | | | | | |

**Table S6.** Baseline characteristics (RCTs)

| **Author** | **Treatment arm** | **Dose** | **No. patients** | **Age mean (SD)** | **Males %** | **Race** | **Comorbidities** | **BMI mean (SD)** | **Smoking status** |
| --- | --- | --- | --- | --- | --- | --- | --- | --- | --- |
| Adam 1982 | Placebo | - | 10 | - | - | - | HTN: 100% | - | - |
| Adam 1982 | Labetalol | 200 | 10 | - | - | - | HTN: 100% | - | - |
| Adam 1982 | Metoprolol | 100 | 10 | - | - | - | HTN: 100% | - | - |
| Adam 1982 | Atenolol | 100 | 10 | - | - | - | HTN: 100% | - | - |
| Adam 1982 | Propranolol | 80 | 10 | - | - | - | HTN: 100% | - | - |
| Hawkins 2009 | Placebo | - | 13 | 68.7 | 77 | - | HF 100%,  Angina: 31%  MI: 46%  AF: 23% | 26.9 (4.4) | Former smokers: 100% |
| Hawkins 2009 | Bisoprolol | Started at 1.25mg,  increased to 10mg | 14 | 72.8 | 64 | - | HF: 100%,  Angina: 29%  MI: 36%  AF: 29% | 29.2 (5.6) |  |
| Lainscak 2011 | Bisoprolol | Mean 6.4 g daily | 32 | 72 (8) | 78 | - | HF:100%; IHD:72%; HTN:81%; DM: 31% | 27.8 (3.9) |  |
| Lainscak 2011 | Carvedilol | Mean 47 mg daily | 31 | 73 (9) | 84 | - | HF: 100%  IHD: 65%  HTN: 74% DM: 35% | 26.8 (5.4) |  |
| Dorow 1986 | Placebo |  | 12 | 45.8 (6.4) | 92 | - | CHD: 100% | - | - |
| Dorow 1986 | Atenolol | 100mg | 12 | 45.8 (6.4) | 92 | - | CHD: 100% | - | - |
| Dorow 1986 | Bisoprolol | 20mg | 12 | 45.8 (6.4) | - | - | - | - | - |
| McGavin 1979 | Propranolol | 80 mg | 9 | 63 (4) | 100 | - | - | - | - |
| McGavin 1979 | Metoprolol | 100 mg | 9 | 63 (4) | 100 | - | - | - | - |
| Van Der Woude 2005 | Placebo | - | 15 | 60.5 (7.3) | 87 | - | - | - | - |
| Van Der Woude 2005 | Celiprolol | 200 mg | 15 | 60.5 (7.3) | 87 | - | - | - | - |
| Van Der Woude 2005 | Metoprolol | 100 mg | 15 | 60.5 (7.3) | 87 | - | - | - | - |
| Van Der Woude 2005 | Propranolol | 80 mg | 15 | 60.5 (7.3) | 87 | - | - | - | - |
| Mainguy 2012 | Bisoprolol | 2.5 mg/day for 2 days;  up titrated to 5 mg/day for 2 days. | 27 | 65 (8) | 63 | - | - | 27 (5) | - |
| Mainguy 2012 | Placebo | - | 27 | 65 (8) | 63 | - | - | 27 (5) | - |
| Chang 2010 | Placebo | - | 11 | 65 | 73 | - | - | - | Current smokers: 45 %  Former smokers: 55% |
| Chang 2010 | Propranolol | 80 mg daily | 11 | 65 | 73 | - | - | - |  |
| Chang 2010 | Metoprolol 190 mg open label | 190 mg daily | 11 | 65 | 73 | - | - | - |  |
| Jabbal 2017 | Bisoprolol | 5mg qd | 18 | 65 | 83 | Caucasian : 100% | - | - | Former smokers: 100% |
| Jabbal 2017 | Carvedilol | 12.5mg bid | 18 | 65 | 83 | Caucasian: 100% | - | - |  |
| Sinclair 1979 | Placebo | 0.9 % saline | 10 | 63 | - | - | - | - | Current smokers: 100% |
| Sinclair 1979 | Propranolol | mean 3-8 mg | 10 | 63 | - | - | - | - |  |
| Sinclair 1979 | Metoprolol | mean 7-6 mg | 10 | 63 | - | - | - | - |  |
| Chester 1981 | Placebo |  | 13 | 53.7 (5.3) | 100 | - | - | - | - |
| Chester 1981 | Propranolol | 40mg | 13 | 53.7 (5.3) | 100 | - | - | - | - |
| Ranchod 1982 | Placebo |  | 15 | 39 | - | - | - | - | Current smokers: 100% |
| Ranchod 1982 | Propranolol | 140 mg per day | 15 | 39 | - | - | - | - |  |
| Ranchod 1982 | Atenolol | 100 mg per day | 15 | 39 | - | - | - | - |  |
| Excluded from NMA |  |  |  |  |  |  |  |  |  |
| Butland 1980 | Placebo | - | 10 | 61 (11) | 60 | - | - | - | - |
| Butland 1980 | Atenolol | 100 mg daily for 4 weeks | 10 | 62 (11) | 60 | - | - | - | - |
| Butland 1980 | Metoprolol | 100 mg daily for 4 weeks | 10 | 63 (11) | 60 | - | - | - | - |
| Dransfield 2019 | Metoprolol | After adjustment: 25 mg, 50 mg, or 100 mg | 268 | 65.2 (7.5) | 53.7 | White: 66.4 % Black: 31 % Other: 2.6 % | CAD,: 14.9%, DM:16.4%, HTN: 44.0% | 26.9 (6.9) | Current smokers: 35.4% |
| Dransfield 2019 | Placebo |  | 264 | 64.8 (7.9) | 53.4 | White: 73.5 % Black: 22.7% Other: 3.8 % | CAD:14.8%, DM: 15.2%, HTN: 48.9% | 27.4 (6.1) | Current smokers: 26.9% |
| AF, atrial fibrillation, bid, twice a day; CAD, coronary artery disease; CHD, coronary heart disease; DM, diabetes mellitus; g, gram; HF heart failure; HTN, hypertension; IHD, ischemic heart disease; MI, myocardial infarction; Mg, milligram; qd, once a day | | | | | | | | | |

**Table S7.** FEV1 measurements -RCTs

| **Author** | **No. arms** | **Treatment arm** | **Timepoint** | **No. randomized** | **Baseline FEV1 (SD)** | **Follow-up FEV1 (SD)** | **Mean change in FEV1 (SD)** | **SE for mean change in FEV1** |
| --- | --- | --- | --- | --- | --- | --- | --- | --- |
| Adam 1982 | 5 | Placebo | 3.5h | 10 | 1.69 (N/R) | 1.6 (0.03) | -0.09 (N/R) | 0.031 |
| Adam 1982 | 5 | Labetalol | 3.5h | 10 | 1.69 (N/R) | 1.7 (0.033) | 0.01 (N/R) | 0.033 |
| Adam 1982 | 5 | Metoprolol | 3.5h | 20 | 1.69 (N/R) | 1.6 (0.047) | -0.09 (N/R) | 0.047 |
| Adam 1982 | 5 | Atenolol | 3.5h | 20 | 1.69 (N/R) | 1.54 (0.045) | -0.15 (N/R) | 0.045 |
| Adam 1982 | 5 | Propranolol | 3.5h | 10 | 1.69 (N/R) | 1.46 (0.06) | -0.23 (N/R) | 0.057 |
| Hawkins 2009 | 2 | Placebo | 4 months | 13 | 1.26 (0.42) | 1.38 (0.026) | 0.12 (0.21) | 0.116 |
| Hawkins 2009 | 2 | Bisoprolol | 4 months | 14 | 1.37 (0.42) | 1.3 (0.026) | -0.07 (0.08) | 0.11 |
| McGavin 1979 | 2 | Propranolol | 1h | 9 | 1.25 (0.49) | 1.1 (0.48) | -0.15 | N/R |
| McGavin 1979 | 2 | Metoprolol | 1h | 9 | 1.15 (0.43) | 1.13 (0.53) | -0.02 (N/R) | N/R |
| McGavin 1979 | 2 | Propranolol | 6h | 9 | 1.25 (0.49) | 1.1 (0.45) | -0.15 (N/R) | 0.22 |
| McGavin 1979 | 2 | Metoprolol | 6h | 9 | 1.15 (0.43) | 1.19 (0.38) | 0.04 (N/R) | 0.19 |
| Van Der Woude 2005 | 4 | Placebo | 4 days | 15 | 2.41 (0.36) | 2.24 (0.37) | -0.17 (N/R) | 0.13 |
| Van Der Woude 2005 | 4 | Celiprolol | 4 days | 15 | 2.41 (0.36) | 2.32 (0.29) | -0.09 (N/R) | 0.12 |
| Van Der Woude 2005 | 4 | Metoprolol | 4 days | 15 | 2.41 (0.36) | 2.16 (0.36) | -0.25 (N/R) | 0.13 |
| Van Der Woude 2005 | 4 | Propranolol | 4 days | 15 | 2.41 (0.36) | 2.08 (0.31) | -0.33 (N/R) | 0.12 |
| Lainscak | 2 | Bisoprolol | 3-4 months | 32 | 1.561 (0.414) | 1.698 (0.519) | 0.137 (N/R) | 0.12 |
| Lainscak | 2 | Carvedilol | 3-4 months | 31 | 1.704 (0.484) | 1.734 (0.548) | 0.03 (N/R) | 0.13 |
| Mainguy 2012 | 2 | Bisoprolol | 14 days | 27 | 1.4 (0.45) | 1.35 (0.44) | -0.05 (N/R) | 0.12 |
| Mainguy 2012 | 2 | Placebo | 14 days | 27 | 1.4 (0.45) | 1.39 (0.45) | -0.01 (N/R) | 0.12 |
| Chang 2010 | 4 | Placebo | 7-10 days | 14 | 1.64 (0.53) | 1.6 (0.53) | -0.04 (N/  R) | 0.2 |
| Chang 2010 | 4 | Metoprolol 95 mg | 7-10 days | 14 | 1.64 (0.53) | 1.54 (0.58) | -0.1 (N/R) | N/R |
| Chang 2010 | 4 | Propranolol | 7-10 days | 14 | 1.64 (0.53) | 1.48 (0.5) | -0.16 (N/R) | 0.19 |
| Chang 2010 | 4 | Metoprolol 190 | 7-10 days | 14 | 1.64 (0.53) | 1.59 (0.55) | -0.05 (N/R) | 0.24 |
| Jabbal 2017 | 2 | Bisoprolol | 6 weeks | 25 | 1.5 (0.7) | 1.34 (0.67) | -0.16 (N/R) | 0.19 |
| Jabbal 2017 | 2 | Carvedilol | 6 weeks | 25 | 1.5 (0.7) | 1.26 (0.7) | -0.24 (N/R) | 0.198 |
| Sinclair 1979 | 3 | Placebo | 1 h | 10 | 1.34 (0.56) | 1.3 (0.49) | -0.04 (N/R) | 0.235 |
| Sinclair 1979 | 3 | Propranolol | 1 h | 10 | 1.33 (0.62) | 1.13 (0.27) | -0.2 (N/R) | 0.213 |
| Sinclair 1979 | 3 | Metoprolol | 1 h | 10 | 1.29 (0.48) | 1.22 (0.3) | -0.07 (N/R) | 0.179 |
| Chester 1981 | 2 | Placebo | 3h | 13 | 1.55 (0.85) | 1.47 (0.45) | -0.08 (N/R) | 0.266 |
| Chester 1981 | 2 | Propranolol | 3h | 13 | 1.55 (0.85) | 1.39 (0.5) | -0.16 (N/R) | 0.273 |
| Dorow 1986 | 3 | Placebo | 4h | 12 | 1.59 (0.19) | 1.582 (0.34) | -0.008 (N/R) | 0.112 |
| Dorow 1986 | 3 | Atenolol | 4h | 12 | 1.59 (0.19) | 1.42 (0.05) | -0.167 (N/R) | 0.056 |
| Dorow 1986 | 3 | Bisoprolol | 4h | 12 | 1.59(0.19) | 1.61 (0.54) | 0.025(N/R) | 0.165 |
| Ranchod 1982 | 3 | Placebo | 2h | 15 | 2.42 (0.44) | 2.39 (0.45) | -0.03 (N/R) | 0.162 |
| Ranchod 1982 | 3 | Propranolol | 2h | 15 | 2.27 (0.44) | 2.15 (0.4) | -0.12 (N/R) | 0.153 |
| Ranchod 1982 | 3 | Atenolol | 2h | 15 | 2.38 (0.19) | 2.25 (0.05) | -0.13 (N/R) | 0.05 |
| SD, standard deviation; N/R, not reported; FEV1, forced expiratory volume in 1 second; No., number | | | | | | | | |

**Table S8.** Network meta-analysis results - league table

| Atenolol | 0.0426 (-0.172, 0.265) | -0.0552 (-0.426, 0.325) | 0.205 (-0.104, 0.517) | 0.185 (0.0404, 0.338) | 0.0809 (-0.0635, 0.231) | -0.0443 (-0.184, 0.1) | 0.0986 (-0.0151, 0.224) |
| --- | --- | --- | --- | --- | --- | --- | --- |
| -0.0426 (-0.265, 0.172) | Bisoprolol | -0.0984 (-0.402, 0.207) | 0.162 (-0.198, 0.518) | 0.143 (-0.0964, 0.377) | 0.0392 (-0.198, 0.271) | -0.0867 (-0.322, 0.145) | 0.057 (-0.144, 0.256) |
| 0.0552 (-0.325, 0.426) | 0.0984 (-0.207, 0.402) | Carvedilol | 0.26 (-0.213, 0.73) | 0.242 (-0.15, 0.623) | 0.137 (-0.252, 0.516) | 0.0111 (-0.376, 0.391) | 0.155 (-0.212, 0.517) |
| -0.205 (-0.517, 0.104) | -0.162 (-0.518, 0.198) | -0.26 (-0.73, 0.213) | Celiprolol | -0.0186 (-0.333, 0.297) | -0.123 (-0.423, 0.178) | -0.248 (-0.549, 0.0519) | -0.105 (-0.403, 0.196) |
| -0.185 (-0.338, -0.0404) | -0.143 (-0.377, 0.0964) | -0.242 (-0.623, 0.15) | 0.0186 (-0.297, 0.333) | Labetalol | -0.105 (-0.258, 0.0485) | -0.231 (-0.383, -0.074) | -0.0872 (-0.22, 0.0565) |
| -0.0809 (-0.231, 0.0635) | -0.0392 (-0.271, 0.198) | -0.137 (-0.516, 0.252) | 0.123 (-0.178, 0.423) | 0.105 (-0.0485, 0.258) | Metoprolol | -0.126 (-0.267, 0.0183) | 0.0178 (-0.11, 0.152) |
| 0.0443 (-0.1, 0.184) | 0.0867 (-0.145, 0.322) | -0.0111 (-0.391, 0.376) | 0.248 (-0.0519, 0.549) | 0.231 (0.074, 0.383) | 0.126 (-0.0183, 0.267) | Propranolol | 0.143 (0.0175, 0.275) |
| **-0.0986 (-0.224, 0.0151)** | **-0.057 (-0.256, 0.144)** | **-0.155 (-0.517, 0.212)** | **0.105 (-0.196, 0.403)** | **0.0872 (-0.0565, 0.22)** | **-0.0178 (-0.152, 0.11)** | **-0.143 (-0.275, -0.0175)** | Placebo |

**Table S9.** SUCRA ranking probability of being the best treatment (improvement in FEV1) in patients with COPD (main analysis)

| **Beta-blocker** | **Probability best** |
| --- | --- |
| Labetalol | 86% |
| Celiprolol | 80% |
| Placebo | 63% |
| Metoprolol | 56% |
| Bisoprolol | 45% |
| Atenolol | 28% |
| Carvedilol | 26% |
| Propranolol | 16% |

**Table S10.** Mortality estimates of beta-blocker versus no beta-blocker use, from individual studies

| **Author** | **Comparison** | **Mean (SD)/median [IQR] follow-up (months)** | **All-cause mortality HR [95% CI]** | **Covariates adjusted for in analysis** |
| --- | --- | --- | --- | --- |
| Rutten, 2010 | BB vs. no BB | 86.4 (NR) | 0.68 [0.56 -0.83] | Age, sex, smoker, diabetes, HTN, CVD, pulmonary drugs, referral to pulmonologist |
| Rutten, 2010 | Cardioselective BB vs. no BB | 86.4 (NR) | 0.67 [0.55 - 0.83] | Age, sex, smoker, diabetes, HTN, CVD, pulmonary drugs, referral to pulmonologist |
| Rutten, 2010 | Non-cardioselective vs. no BB | 86.4 (NR) | 0.82 [0.61-1.1] | Age, sex, smoker, diabetes, HTN, CVD, pulmonary drugs, referral to pulmonologist |
| Short, 2011 | BB vs. no BB | 52 (27) | 0.78 [0.67 – 0.92] | Age, sex, cardiovascular and respiratory hospital admissions, DM, smoking, cardiac drug use (aspirin, statins, CCBs, ACEis), FEV1, resting arterial oxygen saturation, deprivation index |
| Quint, 2013 | BB vs. no BB | 34.8 [1.07 – 86.4] | 0.5 [0.36 – 0.69] | Age, sex, smoking history, angina, HTN, dyslipidaemia, PAD, CVD, HF, DM, MI, frequent exacerbations, diuretics before MI, anti-arrhythmia drugs, ACEi, nitrates and CCBs, anti-platelets and statins |
| Zeng, 2013 | BB vs. no BB | 22.2 [NR] | 0.96 [0.40 – 2.29] | Age, BMI, blood pressure, HR, biochemical markers, echocardiographic parameters, COPD severity, NYHA classification, current smoking status, comorbidities, prescribed drug use |
| Bhatt 2016 | BB vs. no BB | 25.2 [NR] | 0.85 [0.54 – 1.32] | Age, sex, race, smoking burden in pack-years, BMI, CAD, HF, CAC, FEV1, %emphysema on CT, respiratory medications |
| Mentz, 2013 | Cardioselective BB vs. no BB | 2 (NR) | 0.53 [0.25 – 1.13] | Age, sex, cause of admission, depression, liver disease, weight, systolic blood pressure, lower extremity edema, serum sodium, serum creatinine, statin use, arrhythmias, HTN, hyperlipidemia, CAD, ICD or pacemaker, DBP |
| Mentz, 2013 | Non-cardioselective BB vs. no BB | 2 (NR) | 0.47 [0.25 - 0.89] | Age, sex, depression, liver disease, weight, SBP, lower extremity edema, serum sodium, serum creatinine, statin use, cause of admission, arrhythmias, HTN, hyperlipidemia, CAD, ICD or pacemaker, DBP |
| Gottlieb, 1998 | BB vs. no BB | 24 (NR) | 0.6 [0.57 - 0.63] | Unadjusted |
| Sin, 2002 | BB vs. no BB | 21 (NR) | 0.78 [0.63 - 0.95] | Age, sex, CCI, HTN, IDH, propensity scores for BB, use of other medications for HF |
| Ekstrom, 2013 | BB vs. no BB | 13 [NR] | 1.19 [1.04 - 1.37] | Age, sex, BMI, WHO performance status, resting blood gas tensions breathing air, comorbidities, concomitant medication |
| Coiro, 2016 | BB vs. no BB | 20 [NR] | 0.73 [0.6 - 0.9] | Age, sex, smoking habit, Killip class >=3, MI, HF, HTN, renal failure, AF, PAD, DM, CVD, SBP, DBP, HR, eGFR, LVEF, digoxin, ACE/ARB, diuretics, aspirin, CCB, statins |
| Staszewsky, 2016 | BB vs. no BB | 48 [NR] | 0.74 [0.64 - 0.84] | Age, sex, comorbidities |
| Su, 2016 | Carvedilol, high dose (nonselective) vs. no BB | 52 (NR) | 0.81 [0.56 - 1.18] | Age, sex, severity of COPD and HF, DM, dysrhythmia, ischemic stroke, intracranial hemorrhage, HTN, IHD, CKD, liver cirrhosis |
| Su, 2016 | Bisoprolol, high dose (cardioselective) vs. no BB | 52 (NR) | 0.4 [0.26 - 0.63] | Age, sex, severity of COPD and HF, DM, dysrhythmia, ischemic stroke, intracranial hemorrhage, hypertension, IHD, CKD, liver cirrhosis |
| Su, 2016 | Metoprolol, high dose (cardioselective) vs. no BB | 52 (NR) | 0.36 [0.09 - 1.43] | Age, sex, severity of COPD and HF, DM, dysrhythmia, ischemic stroke, intracranial hemorrhage, HTN, IHD, CKD, liver cirrhosis |
| Kubota, 2015 | BB vs. no BB | 33.9 (NR) | 0.46 [0.19 - 1.11] | Age, sex, BMI, HTN, AF, BB, BNP, LVEF, ACEI or ARB, GOLD stage 3-4, history of COPD exacerbation, inhaled tiotropium |
| Hawkins, 2009 | BB vs. no BB | 24.7 (NR) | 0.74 [0.68 - 0.8] | Unadjusted |
| Ellingsen, 2020 | BB vs. no BB | study period 10 years (follow up 64,306 person-years, no other details) | 0.86 [0.76 - 0.97] | Age, sex, education, marital status, income, pneumonia, HF, MI, IHD, stroke, HTN, DM, osteoporosis, depression, asthma, exacerbations |
| Rodriguez-Manero, 2019 | BB vs. no BB | 23 (3.3) | 0.62 [0.38 - 0.99] | Age, sex, HF, HTN, thromboemoblic event, vascuopathy, DM, AF, dementia, oral anticoagulants, antiplatelet, ACEi/ARBs, digoxin |
| Su 2019b | cardioselective BB vs. no BB | 112 (NR) | 0.72 [0.71 - 0.72] | Age, sex, income level, comorbidities, exacerbation frequency of COPD and HF, CCI, urbanization level, SABD, LABA, ICS, ICS/LABA, LAMA, ACEi, ARB, aldosterone, digoxin, statins |
| Su 2019b | nonselective BB vs. no BB | 112 (NR) | 0.92 [0.92 - 0.93] | Age, sex, income level, comorbidities, exacerbation frequency of COPD and HF, CCI, urbanization level, SABD, LABA, ICS, ICS/LABA, LAMA, ACEi, ARB, aldosterone, digoxin, statins |
| Su 2019 | BB vs. no BB (patients receiving PCI/CABG) | Overall survival | 0.87 [0.82 -0.92 ] | Adjusted with IPTW on covariates: age, sex, socioeconomic status, length of stay for the index acute MI, comorbidities, previous outpatient treatment for COPD, inpatient treatments, complications of acute MI during hospitalization, other outpatient prescriptions |
| Su 2019 | BB vs. no BB (not receiving PCI/CABG) | Overall survival | 0.94 [0.85 – 1.04] | Adjusted with IPTW on covariates: age, sex, socioeconomic status, length of stay for the index acute MI, comorbidities, previous outpatient treatment for COPD, inpatient treatments, complications of acute MI during hospitalization, other outpatient prescriptions |
| Wang 2019 | Cardioselective vs. no BB | 96 (NR) | 0.93 [0.89 – 0.98] | Age, sex, HTN, DM, PVD, HF, previous CVA, ESRF, AF, MI, PCI, antiplatelet, ACEi, ARB, statin, CCB, xanthins, corticosteroids, SAMA, LAMA |
| Wang 2019 | Non-cardioselective vs. no BB | 96 (NR) | 0.98 [0.94 – 1.02] | Age, sex, HTN, DM, PVD, HF, previous CVA, ESRF, AF, MI, PCI, antiplatelet, ACEi, ARB, Statin, CCB, xanthins, corticosteroids, SAMA, LAMA |
| Wang 2019 | BB vs. no BB | 96 (NR) | 0.97 [0.93 - 1] | Age, sex, HTN, DM, PVD, HF, previous CVA, ESRF, AF, MI, PCI, antiplatelet, ACEi, ARB, Statin, CCB, xanthins, corticosteroids, SAMA, LAMA |
| Scrutinio, 2019 | BB vs. no BB | 24 (NR) | 0.66 [0.53 – 0.83] | Age, sex, DM, HF-related hospitalizations in the 6 months preceding the index event, symptoms severity at admission, admission SBP, use of inotropes during hospitalization, LVEF, eGFR, NT-proBNP, hemoglobin, sodium levels |
| Van Gestel , 2008 | BB vs. no BB | 120 (NR) | 0.73 (0.6 – 0.88) | Age, sex, HTM, hypercholesterolemia, DM, renal dysfunction, smoking status, BMI, type of surgery, year of surgery, CVD history, a composite variable of statins, aspirin and ACEi |
| ACEis, angiotensin-converting-enzyme inhibitors; ARB, angiotensin receptor blockers; AF, atrial fibrillation; BB, beta-blockers; BMI, body mass index; CAC, coronary artery calcification; CCB, calcium channel blocker; CCI, Charlson Comorbidy Index; CT, computed tomography; CVA, cerebrovascular accident; CVD, cardiovascular disease; DBP, diastolic blood pressure; DM diabetes mellitus; eGFR, estimated glomerular filtration rate; ESRF, end stage renal failure; FEV1, forced expiratory volume in 1 second; GOLD, Global Initiative for Chronic Obstructive Lung Disease; HF, heart failure; HR, heart rate; HTN, hypertension; ICD, implantable cardioverter-defibrillator; IDH, intradialytic hypotension; IPTW; inverse probability treatment weighting; LVEF, left ventricular ejection fraction; LAMA, long-acting muscarinic antagonist; MI, myocardial infarction; NT-proBNP, N-terminal pro-brain natriuretic peptide; NYHA, New York Heart Association; PAD, peripheral artery disease; PCI, percutaneous coronary intervention; PVD, peripheral vascular disease; SABD, short acting bronchodilator; SAMA, short-acting muscarinic antagonist; SBP, systolic blood pressure; WHO, World Health Organization | | | | |

**Table S11.** All-cause hospitalization results

| **Author** | **Study design** | **Comparison** | **Follow-up** | **Estimate (95 CI)** | **Covariates adjusted for in analysis** | **Notes** |
| --- | --- | --- | --- | --- | --- | --- |
| Sessa, 2018a ; Denmark | Observational | Non-cardioselective BB vs. cardioselective BB | 7567  person-years | HR: 1.01 (0.93–1.10) | Age, year of inclusion in the cohort, vital status, pharmacological treatments, comorbidities | Only study presenting results for non-cardiovascular BB vs. cardioselective BB; |
| Brooks, 2007 | Observational | Cardioselective BB vs. no BB | 3611  patient-years | RR: 0.64 (0.43–0.96) |  |  |
| Farland, 2013 | Observational | Cardioselective BB vs. non-cardioselective BB | 1 year | OR: 1.41 (0.95 - 2.09) | Age, sex, smoking status, angina, MI, CABG, PCI, AF, HF, PAD, DM, primary care vs. specialist, inhaled anticholinergic, inhaled LABA, inhaled corticosteroid, xanthine derivative, ACEI, ARB, aldosterone antagonist, clopidogrel, statin, thiazide diuretic, loop diuretic, nitrate, CCB, digoxin, vitamin K antagonist) |  |
| ACEI, angiotensin-converting-enzyme inhibitors; ARB, angiotensin receptor blockers; AF, atrial fibrillation; BB, beta-blockers; CABG, coronary artery bypass grafting; CCB, calcium channel blockers; DM, diabetes mellitus; HF, heart failure; HTN, hypertension; LABA, long-acting beta-agonist; PCI, percutaneous coronary intervention; MI, myocardial infarction; PAD, peripheral arterial disease; RR, relative risk; CI, confidence interval; OR, odds ratio; HR, hazard ratio | | | | | | |

**Table S12.** SGRQ results

| **Author, year** | **Study design** | **Treatment arm** | **Timepoint** | **Baseline SGRQ (SD) [SE] in meters** | **Follow-up SGRQ (SD)** | **Mean change (SD)** |
| --- | --- | --- | --- | --- | --- | --- |
| Jabbal, 2017 | RCT | Bisoprolol | 6 weeks | 33 (24–42) | 36 (28–44) | 3 (N/R) |
| Jabbal, 2017 | RCT | Carvedilol | 6 weeks | 33 (24–42) | 36 (26–45) | 3 (N/R) |
| Dransfield 2019 | RCT | Metoprolol | 52 weeks | - | - | Metroprolol vs. placebo, baseline to timepoint: 0.77 (-1.38, 2.92) |
| Dransfield 2019 | RCT | Placebo | 52 weeks | - | - | - |
| Maltais, 2018 | Observational | No BB | 52 weeks | 43.60 [0.28) | 37.90 [0.19] | BB vs. no BB, baseline to timepoint: -0.60 (-1.810 to 0.602) |
| Maltais, 2018 | Observational | BB | 52 weeks | 43.58 [0.76) | 37.29 [0.58] |  |
| BB, beta-blockers; RCT, randomized controlled trial; SGRQ, St. George Respiratory Questionnaire; SD, standard deviation; SE, standard error; N/R, not reported | | | | | | |

**Table S13.** 12MWT results

| **Author, year** | **Study design** | **Treatment arm** | **Timepoint** | **Study design** | **Baseline 12MWT (SD) in meters** | **Follow-up 12MWT (SD)** | **Mean change (SD)** |
| --- | --- | --- | --- | --- | --- | --- | --- |
| Butland 1980, | RCT | Placebo | 4 weeks | RCT | - | 12MWT post mean: 715 (225) | - |
|  | RCT | Atenolol | 4 weeks | RCT | - | 675 (227) | - |
|  | RCT | Metoprolol | 4 weeks | RCT | - | 680 (228) | - |
| McGavin, 1979 | RCT | Propranolol | 6 h | RCT | 1058 (255) | 1158 (162) | 100 (N/R) |
|  | RCT | Metoprolol | 6 h | RCT | 1059 (314) | 1154 (199) | 95 (N/R) |
| 12MWT, 12-minute walking -test; RCT, randomized controlled trial, N/R, not reported; SD, standard deviation | | | | | | | |

**Table S14.** 6MWT results

| **Author, year** | **Study design** | **Treatment arm** | **Timepoint** | **Study design** | **Baseline 6MWT (SD) in meters** | **Follow-up 6MWT (SD)** | **Mean change (SD)** |
| --- | --- | --- | --- | --- | --- | --- | --- |
| Jabbal, 2017 | RCT | Bisoprolol | 6 weeks | RCT | 495 (101) | 469 (101) | -26 (N/R) |
|  | RCT | Carvedilol | 6 weeks | RCT | 495 (101) | 474 (125) | -25 (N/R) |
| Dransfield 2019 | RCT | Metoprolol | 52 weeks | RCT | - | - | Metoprolol vs. placebo, baseline to timepoint: -5.77 (95% CI –21.59, 10.06) |
|  | RCT | Placebo | 52 weeks | RCT | - | - | - |
| 6MWT, 6-minute walking test; RCT, randomized controlled trial; N/R, not reported; SD, standard deviation; CI, confidence intervals | | | | | | | |

**Table S15.** SF-36 results

| **Author, year** | **Study design** | **Treatment arm** | **N** | **Timepoint** | **Results* notes** |
| --- | --- | --- | --- | --- | --- |
| van Gestel 2009 | Observational | Beta-blocker | 191 | 6.4 years (2.9 - 9.3) | No significant associations between beta-blockers  and the individual domains of the SF-36 in patients  (PF: OR 1.36; 95% CI 0.72–2.61, RP: OR 1.55;  95% CI 0.78–3.06, BP: OR 1.00; 95% CI 0.52–1.94, GH: OR  1.27; 95% CI 0.67–2.41, VT: OR 1.29; 95% CI 0.68–2.44, SF: OR 1.59; 95% CI 0.87–2.92, RE: OR 1.00; 95% CI  0.50–1.97, MH: OR 1.15; 95% CI 0.62–2.14)  Beta-blocker therapy at follow-up was not associated with  impaired health status (PF: OR 1.27; 95% CI 0.72–2.27, RP:  OR 1.66; 95% CI 0.92–2.98, BP: OR 0.96; 95% CI 0.55–1.69,  GH: OR 1.50; 95% CI 0.84–2.66, VT: OR 1.22; 95% CI  0.69–2.14, SF: OR 1.34; 95% CI 0.78–2.29, RE: OR 1.27; 95%  CI 0.70–2.30, MH: OR 1.57; 95% CI 0.89–2.75) |
| van Gestel 2009 | Observational | No beta-blocker | 135 | 6.4 years (2.9 - 9.3) |  |
| *Results not presented per treatment arm or per overall SF-36; The SF-36 questionnaire has 8 domains: physical functioning (PF), role physical (RP), bodily pain (BP), general health (GH), vitality (VT), social functioning (SF), role emotional (RE) and mental health (MH).  OR, odds ratio; CI, confidence intervals; SF-36, Short-Form Health Survey Questionnaire | | | | | |

**Table S16**. Risk of bias assessment, observational studies

| **Author, year** | **Bias due to confounding** | **Bias in selection of participants into study** | **Bias in classification of interventions** | **Bias due to departures from intended interventions** | **Bias due to missing data** | **Bias in measurement of outcomes** | **Bias in selection of the reported result** | **Overall** |
| --- | --- | --- | --- | --- | --- | --- | --- | --- |
| Bhatt 2016 | Low | Moderate | Moderate | Moderate | NI | Moderate | Low | Moderate |
| Brooks, 2007 | Moderate | Low | Moderate | NI | NI | Low | Low | Moderate |
| Coiro, 2016 | Low | Low | Moderate | Low | Low | Low | Low | Low |
| Ekstrom, 2013 | Moderate | Moderate | Low | NI | Low | Low | Low | Moderate |
| Ellingsen, 2020 | Moderate | Moderate | Low | NI | Low | Low | Low | Moderate |
| Gottlieb, 1998 | NI | NI | Moderate | NI | Moderate | Low | Low | NI |
| Hawkins, 2009 | Moderate | Moderate | Low | Low | Low | Low | Low | Moderate |
| Maltais, 2018 | Low | Moderate | Low | Low | Low | Low | Low | Low |
| Mentz, 2013 | Low | Moderate | Moderate | NI | Low | Low | Low | Moderate |
| Quint, 2013 | Moderate | Moderate | Moderate | NI | Low | Low | Low | Moderate |
| Rodriguez-Manero, 2019 | Moderate | Moderate | Serious | NI | Low | Low | Low | Moderate |
| Rutten, 2010 | Moderate | Serious | Low | NI | Low | Low | Moderate | Moderate |
| Scrutinio, 2019 | Moderate | Moderate | Moderate | NI | Low | Low | Low | Moderate |
| Short, 2011 | Moderate | Moderate | Moderate | NI | NI | Low | Low | Moderate |
| Sin, 2002 | Moderate | Moderate | Low | NI | NI | Low | Low | Moderate |
| Staszewsky, 2016 | Moderate | Moderate | Moderate | NI | NI | Moderate | Low | Moderate |
| Su 2019 | Moderate | Moderate | Moderate | NI | Low | Low | Low | Moderate |
| Su, 2016 | Moderate | Moderate | Moderate | NI | NI | Low | Low | Moderate |
| Su, 2019b | Moderate | Moderate | Moderate | NI | Low | Low | Low | Moderate |
| van Gestel, 2008 | Moderate | Moderate | Moderate | NI | Low | Low | Low | Moderate |
| Wang 2019 | Moderate | Moderate | Moderate | NI | Low | Low | Low | Moderate |
| Zeng, 2013 | Serious | Serious | Moderate | NI | NI | Moderate | Moderate | Serious |
| Sessa 2018a | Moderate | Low | Moderate | NI | Low | Low | Low | Moderate |
| van Gestel 2009 | Low | Low | Moderate | NI | Low | Low | Low | Moderate |
| Farland, 2013 | Moderate | Moderate | Moderate | NI | NI | Moderate | Low | Moderate |
| Rasmussen, 2020 | Moderate | Low | Moderate | NI | Low | Low | Low | Moderate |

**Table S17.** GRADE assessment for effect of beta-blocker versus placebo or lack of beta-blocker, on mortality and quality of life outcomes

|  | Quality assessment | | | | | |  | Effect | Quality |
| --- | --- | --- | --- | --- | --- | --- | --- | --- | --- |
| Outcome | Number of studies | Study design | Risk of bias | Inconsistency | Indirectness | Imprecision | Likelihood of publication bias |  |  |
| Mortality | 21 | Observational | Serious | Serious | Not serious | Serious | Suspected | Most studies shows relative reduction of mortality with bb use. | Very low (1, 2, 4, 5) |
| All-cause hospitalization | 3 | Observational | Not serious | Serious | Serious | Serious | Suspected | No significant differences associated with bb | Very low (2, 3, 4, 5) |
| SGRQ | 2 | RCT | Serious | Not serious | Serious | Serious | Suspected | No significant differences associated with bb | Very low (1, 3, 4, 5) |
| SGRQ | 1 | Observational | Not serious | Serious | Serious | Serious | - | No significant differences associated with bb | Very low (2, 3, 4) |
| 12MWT | 2 | RCT | Not serious | Serious | Serious | Serious | - | No significant differences between metoprolol vs propranolol | Very low (2, 3, 4) |
| 6MWT | 2 | RCT | Serious | Not serious | Serious | Serious | Suspected | No significant differences associated with bb | Very low (1, 3, 4, 5) |
| SF-36 | 1 | Observational | Not serious | Serious | Serious | Serious |  | No significant differences associated with bb | Very low (2, 3, 4) |

1. Study design – based on risk of bias; downgrade if serious risk of bias rating for any study
2. Inconsistency – widely differing estimates of effects
3. Indirectness - dissimilarity with inclusion criteria (based on PICOs)
4. Imprecision – estimate of effects includes the null effect for the largest studies
5. Likelihood of publication bias - downgrade if search not comprehensive; likelihood that negative results have not been published

**Table S18.** Grade assessment for effect of beta-blocker therapy on AECOPD

|  |  | Quality assessment | | | | | |  |  | Quality |
| --- | --- | --- | --- | --- | --- | --- | --- | --- | --- | --- |
| Outcome | Participants | Number of studies | Study design | Risk of bias | Inconsistency | Indirectness | Imprecision | Publication bias | Relative effect (95% CI) |  |
| AECOPD | 27717 | 5 | Observational | Not serious | Not serious | Not serious | Not serious | N/A |  | Low (1) |
| AECOPD | 532 | 1 | RCT | Not serious | Serious | Not serious | Not serious | N/A | 1.05 (0.84 1.32) | Moderate (2) |

Reasons for downgrading:

1. Study design
2. Inconsistency – overall quality derived from a single RCT with a small sample size
3. Indirectness
4. Imprecision

**Table S19**. GRADE assessment from each pair-wise comparison within the NMA network (FEV1 analysis)

| Comparison | Direct  evidence | | Indirect evidence | | NMA estimate | |
| --- | --- | --- | --- | --- | --- | --- |
|  | MD (95% CrI) | Quality of evidence | MD (95% CrI) | Quality of evidence | MD (95% CrI) | Quality of evidence |
| Bisoprolol vs Atenolol | 0.19 (-0.15, 0.56) | Moderate (2) | -0.048 (-0.32, 0.25) | Moderate (2) | 0.039 (-0.17, 0.25) | Moderate |
| Atenolol vs. Labetalol | -0.185 (-0.338, -0.0404) | Moderate (2) | - | - | -0.185 (-0.338, -0.0404) | Moderate |
| Metoprolol vs Atenolol | 0.060 (-0.12, 0.26) | Moderate (2) | 0.14 (-0.15, 0.42) | Moderate (2) | 0.083 (-0.067, 0.24) | Moderate |
| **Atenolol vs. Placebo** | **-0.0986 (-0.224, 0.0151)** | **Moderate (2)** | **-** | **-** | **-0.098 (-0.22, 0.014)** | **Moderate** |
| Propranolol vs. Atenolol | -0.059 (-0.24, 0.12) | Moderate (2) | 0.070 (-0.28, 0.42) | Moderate (2) | -0.050 (-0.19, 0.098) | Moderate |
| Bisoprolol vs. Carvedilol | -0.0984 (-0.207, 0.402) | Low (1, 2) | - | - | -0.0984 (-0.207, 0.402) | Low |
| **Bisoprolol vs. Placebo** | **-0.057 (-0.256, 0.144)** | **Low (1, 2)** | **-** | **-** | **-0.057 (-0.256, 0.144)** | **Low** |
| Celiprolol vs. Metoprolol | 0.123 (-0.178, 0.423) | Very low (1, 2) | - | - | 0.123 (-0.178, 0.423) | Very low |
| **Celiprolol vs. Placebo** | **0.105 (-0.196, 0.403)** | **Very low (1, 2)** | **-** | **-** | **0.105 (-0.196, 0.403)** | **Very low** |
| Celiprolol vs. Propranolol | 0.248 (-0.0519, 0.549) | Very low (1, 2) | - | - | 0.248 (-0.0519, 0.549) | Very |
| Labetalol vs. Metoprolol | 0.105 (-0.0485, 0.258) | Very low (1, 2) | - | - | 0.105 (-0.0485, 0.258) | Very low |
| **Labetalol vs. Placebo** | **0.0872 (-0.0565, 0.22)** | **Very low (1, 2)** | **-** | **-** | **0.0872 (-0.0565, 0.22)** | **Very low** |
| Labetalol vs. Propranolol | 0.231 (0.074, 0.383) | Very low (1, 2) | - | - |  | Very low |
| Placebo vs. Metoprolol | 0.017 (-0.13, 0.17) | Moderate (2) | 0.0094 (-0.66, 0.66) | Moderate (2) | 0.017 (-0.11, 0.15) | Moderate |
| Metoprolol vs. Propranolol | 0.126 (-0.0183, 0.267) | Moderate (2) | - | - | 0.126 (-0.0183, 0.267) | Moderate |
| Placebo vs. Propranolol | 0.143 (0.0175, 0.275) | Moderate (2) | - | - | 0.143 (0.0175, 0.275) | Moderate |
| Atenolol vs Carvedilol | - | - | -0.205 (-0.517, 0.104) | Very low(1, 2) | -0.205 (-0.517, 0.104) | Very low |
| Bisoprolol vs Celiprolol | - | - | -0.162 (-0.518, 0.198) | Very low(1, 2) | -0.162 (-0.518, 0.198) | Very low |
| Bisoprolol vs Labetalol | - | - | -0.143 (-0.377, 0.0964) | Very low(1, 2) | -0.143 (-0.377, 0.0964) | Very low |
| Bisoprolol vs Metoprolol | - | - | -0.0392 (-0.271, 0.198) | Very low(1, 2) | -0.0392 (-0.271, 0.198) | Very low |
| **Carvedilol vs placebo** | **-** | **-** | **-0.155 (-0.517, 0.212)** | **Very low(1, 2)** | **-0.155 (-0.517, 0.212)** | **Very low** |
| Celiprolol vs atenolol | - | - | 0.205 (-0.104, 0.517) | Moderate (2) | 0.205 (-0.104, 0.517) | Moderate |
| Celiprolol vs labetalol | - | - | -0.242 (-0.623, 0.15) | Moderate (2) | -0.242 (-0.623, 0.15) | Moderate |
| Celiprolol vs Propranolol |  |  | 0.248 (-0.0519, 0.549) |  | 0.248 (-0.0519, 0.549) |  |
| Labetalol vs. carvedilol | - | - | -0.0186 (-0.333, 0.297) | Very low (1, 2) | -0.0186 (-0.333, 0.297) | Very low |
| Metoprolol vs atenolol |  |  | 0.0809 (-0.0635, 0.231) |  | 0.0809 (-0.0635, 0.231) |  |
| Metoprolol vs Bisoprolol | - | - | 0.0867 (-0.145, 0.322) | Moderate (2) | 0.0867 (-0.145, 0.322) | Moderate |
| **Metoprolol vs Placebo** | **-0.0178 (-0.152, 0.11)** | **Moderate (2)** | **-** | **-** | **-0.0178 (-0.152, 0.11)** | **Moderate** |
| Propranolol vs Bisoprolol | - | - | -0.0867 (-0.322, 0.145) | Moderate (2) | -0.0867 (-0.322, 0.145) | Moderate |
| Propranolol vs carvedilol | - | - | 0.0111 (-0.376, 0.391) | Very low (1, 2) | 0.0111 (-0.376, 0.391) | Very low |
| **Propranolol vs placebo** | **-0.143 (-0.275, -0.0175)** | **High** | **-** | **-** | **-0.143 (-0.275, -0.0175)** | **High** |

GRADE judgments refer not to individual studies but to a body of evidence, and quality, as used in GRADE, means more than risk of bias. A body of evidence (for instance, a number of well-designed and executed trials) may be associated with a low risk of bias, but our confidence in effect estimates may be compromised by a number of other factors (imprecision, inconsistency, indirectness, and publication bias).

**GRADE for FEV1 (network meta-analysis)**

Reasons for downgrading:

**(1) Study limitations:** We downgraded by one level when comparisons were made from at least one study which was rated as a serious or very serious risk of bias

**(2) Imprecision:** We downgraded one level if the estimate in mean change included the null effect. We downgraded one further level if the effect size comes from one study only or there are few events.

**(3) Inconsistency:** We planned to downgrade comparisons with important inconsistency (p<0.01), however all comparisons were consistent (direct and indirect estimates were in agreement), thus we did not downgrade any studies based on this.

**(4) Indirectness:** We ensured there were no treatment modifiers in our analyses by conducting meta-regression, which indicate no implication of covariates assessed. We thus did not downgrade any studies based on this.

**(5) Publication bias:** The comparison-adjusted funnel plot (Figure S3) did not suggest presence of overall publication bias, therefore we did not downgrade for this item.
